# The compounding burden of disability in Mexico: social protection gaps, chronic disease excess, and prospective mortality from national open data sources

**DOI:** 10.3389/fpubh.2026.1847293

**Published:** 2026-07-02

**Authors:** Sofía Gutierrez-Perez, Judith Carolina De Arcos-Jiménez, Mauricio Alfredo Ambriz-Alarcón, Brian Rafael Rubio-Mora, Ana María López-Yáñez, Patricia Noemí Vargas-Becerra, Sahian Alexandra González-Espinosa, José Ángel Castillo-Avila, Nancy Yamile Márquez-Mayorga, Jaime Briseno-Ramirez

**Affiliations:** 1 Comisión Estatal de Derechos Humanos Jalisco, Guadalajara, Mexico; 2 Hospital Civil de Oriente, Tónala, Mexico; 3 Centro Universitario de Tlajomulco, Universidad de Guadalajara, Tlajomulco de Zuñiga, Mexico; 4 Antiguo Hospital Civil de Guadalajara “Fray Antonio Alcalde”, Guadalajara, Mexico; 5 Centro Universitario del Norte, Universidad de Guadalajara, Colotlán, Mexico

**Keywords:** disability, health equity, intersectionality, Mexico, morbidity, mortality, social security

## Abstract

**Background:**

Persons with disabilities (PWD) face disproportionate barriers to social protection and healthcare worldwide, yet comprehensive multi-source evidence linking social security coverage, morbidity, and mortality outcomes remains scarce in Latin America.

**Methods:**

We conducted a cross-sectional, multi-source population-based study with a prospective cohort component, integrating nine nationally representative Mexican data sources (2017–2023) encompassing over 125 million persons. Disability was operationalized using source-specific measures, including Washington Group-based instruments where available. We examined social security coverage disparities, catastrophic health expenditure, chronic disease prevalence, intersectional exclusion, prospective three-year mortality, and geographic overlap with healthcare infrastructure deficits. Survey-weighted logistic regression with progressive adjustment, Benjamini–Hochberg correction for multiple comparisons, and unweighted binomial models for prospective mortality were employed.

**Results:**

Among 6.8 million PWD in the 2020 Census, apparent social security coverage advantages reflected a structural shift to non-contributory programs driven by older age structure; after demographic adjustment the advantage disappeared (OR = 0.99, *p* = 0.30), with a modest residual protective effect after full adjustment (OR = 0.92, *p* < 0.001). Disability compounded exclusion for women (interaction OR = 1.07) and indigenous persons (interaction OR = 1.10; both *p* < 0.001). Catastrophic health expenditure was higher among PWD households (adjusted OR = 2.02). Disease prevalence was significantly higher across all nine conditions assessed (adjusted ORs ranging from 1.46 for diabetes to 2.92 for depressive symptomatology), with less than 5% attenuation after adjustment. Prospective three-year mortality was elevated (adjusted OR = 3.77; 95% CI: 3.02–4.70; risk ratio 3.0), with a severity gradient reaching OR = 7.24. Non-contributory coverage was not associated with lower mortality (OR = 0.89, *p* = 0.32). Geographic analysis ecologically estimated approximately 502,858 triple-vulnerable PWD concentrated in high-marginalization states.

**Conclusions:**

The apparent social security advantage of PWD is an artifact of older age structure and a shift toward non-contributory programs that confer nominal affiliation without a detectable mortality benefit in this older-adult cohort, with disadvantage intensifying for indigenous women with disability. Expanding enrollment alone is likely insufficient: closing this gap requires disability-inclusive coverage that translates affiliation into effective protection.

## 1 Introduction

Disability is a universal human experience that affects an estimated 1.3 billion people (approximately 16% of the global population) according to the most recent estimates from the World Health Organization (1). This figure, which has risen from the 15% estimate reported in the landmark *World Report on Disability* (2), reflects the combined effects of population aging and the epidemiological transition toward noncommunicable diseases (3, 4). The International Classification of Functioning, Disability and Health (ICF) conceptualizes disability not as a fixed attribute of individuals but as the product of interactions between health conditions and contextual factors: environmental barriers, social exclusion, and limited access to services (5). This biopsychosocial framework underpins the Washington Group Short Set on Functioning (WG-SS), which has become the international standard for producing comparable disability data in censuses and national surveys (6). The adoption of the Convention on the Rights of Persons with Disabilities (CRPD) (7), ratified by most countries worldwide including Mexico in 2007, has further established disability as a human rights and development priority embedded in the Sustainable Development Goals (8).

Despite these normative advances, PWD remain disproportionately excluded from social protection systems worldwide. A systematic review of 150 studies from low- and middle-income countries (LMICs) found that 81% reported a significant positive association between disability and economic poverty (9), establishing a well-documented disability–poverty cycle in which disability and deprivation are bidirectionally linked (10–12). Social security coverage, a key mechanism for breaking this cycle, remains inadequate: health insurance coverage among PWD in LMICs is limited and its financial protection effects are “limited and inconclusive” (13, 14). The International Labor Organization reports that PWD earn 12% less per hour on average (26% less in LMICs) and are overrepresented in informal employment lacking social security contributions (15, 16). These gaps are compounded by “extra costs of living” for healthcare, assistive devices, and personal assistance that are associated with catastrophic expenditure (17, 18). When disability intersects with other axes of social stratification (gender, indigenous identity, rural residence), disadvantage becomes super-additive rather than merely cumulative (19–21).

The health consequences of this exclusion are severe and increasingly well documented. An umbrella review synthesizing 58 systematic reviews and 132 meta-analyses found that 86% of pooled estimates demonstrated significant associations between disability and adverse health outcomes (22). PWD experience substantially higher chronic disease prevalence, including elevated odds of diabetes, cardiovascular disease, and chronic kidney disease (23, 24), and depression prevalence ranges from 8% to 66% depending on disability type (25). Multimorbidity, the co-occurrence of two or more chronic conditions, is markedly more common among PWD, further compounding healthcare needs and costs (26). A meta-analysis of 70 prospective cohort studies encompassing 270,571 PWD estimated a pooled hazard ratio of 2.02 (95% CI: 1.77–2.30) for all-cause mortality, with estimates ranging from 1.36 for visual impairment to 3.95 for multiple impairments (27). Long-term follow-up studies have confirmed dose–response gradients, with severe motor disability yielding hazard ratios of 3.67 and severe mental health conditions 3.40 (28, 29). Yet the mechanisms linking social protection gaps to excess morbidity and mortality remain poorly elucidated. PWD face healthcare barriers at multiple levels (financial, physical, attitudinal, and systemic) (30, 31), creating conditions for a “cascade of disparities” in which higher disease burden, inadequate preventive care, and delayed treatment are associated with worse outcomes even when nominal access exists (32). This cascade has been documented primarily in high-income settings, with limited evidence from LMICs (33).

In Latin America and the Caribbean, an estimated 85 million people live with disabilities, yet a recent systematic review found that 76% of quantitative studies on healthcare access for PWD in the region had medium or high risk of bias, and concluded that “large gaps exist in the current evidence” (33). Disability prevalence in the region varies widely, from 4.5% in Trinidad and Tobago to 24.9% in Brazil, reflecting differences in measurement approaches across national censuses (34). In Mexico, the 2020 Census reported that 16.5% of the population experiences some form of disability or functional limitation (35), while approximately 5.7% meet the more restrictive Washington Group threshold for significant disability (36). Despite this substantial population, while Mexico's national poverty evaluation agency (CONEVAL) has published descriptive notes on disability and poverty (37, 38), it does not integrate disability as a cross-cutting dimension in its official multidimensional poverty measurement reports, and the country's General Law for the Inclusion of Persons with Disabilities mandates protections for this population (39). Prior studies using the Mexican Health and Aging Study (ENASEM/MHAS) have examined disability transitions (40), educational gradients in disability onset (41), and the impact of Seguro Popular on disability progression (42), but no study has comprehensively analyzed social security coverage, morbidity burden, and mortality risk among PWD using multiple nationally representative data sources. Educational inequalities in disability linked to social security have been studied in five Latin American countries, but Mexico was not among them (43).

Interpreting these patterns requires familiarity with the structure of Mexico's fragmented health and social protection system. Coverage is delivered through two fundamentally different channels. *Contributory* social insurance is financed by payroll contributions tied to formal employment and provides comprehensive benefit packages: the Instituto Mexicano del Seguro Social (IMSS) covers private-sector salaried workers, while the Instituto de Seguridad y Servicios Sociales de los Trabajadores del Estado (ISSSTE) covers public-sector employees. *Non-contributory* schemes, financed from general taxation, were created to cover the population without formal employment but have historically offered a narrower and less stable set of services; this segment has undergone repeated institutional restructuring, from Seguro Popular (2004–2019) to the Instituto de Salud para el Bienestar (INSABI, 2020–2023) and, most recently, to IMSS-Bienestar (44). A small share of the population holds private insurance, and out-of-pocket spending remains high. Because access to comprehensive contributory coverage is contingent on formal employment, labor-market exclusion is a central mechanism through which disability may shape the quality of social protection—a distinction between nominal affiliation and effective protection that motivates our analysis.

This study addresses these gaps by examining the compounding burden of disability in Mexico across three interconnected dimensions: social protection gaps, chronic disease excess, and prospective mortality. Drawing on nine nationally representative, publicly available data sources encompassing over 125 million persons in expanded population and drawn from the most recent available independent sources (2017–2023), we integrate census microdata, household surveys, health surveys, and administrative records to construct a comprehensive portrait of how disability is associated with differential access to social security, higher morbidity burden, and elevated mortality risk. Specifically, we: (1) quantify social security coverage disparities by disability status, examining the role of age structure and the contributory vs. non-contributory composition of coverage; (2) analyze the intersectional associations of disability, indigenous identity, and gender with social exclusion; (3) assess chronic disease prevalence and healthcare utilization differentials using age-sex and fully adjusted models with multiple comparison corrections; (4) estimate prospective 3-year mortality risk associated with baseline disability severity in a nationally representative cohort of older adults; and (5) map the geographic overlap between disability, social security exclusion, and healthcare infrastructure deficits at the municipal level. To our knowledge, this is the first multi-source, population-level analysis linking disability to social protection, morbidity, and mortality outcomes in Mexico.

## 2 Materials and methods

### 2.1 Study design and setting

This was a cross-sectional, multi-source population-based study with a prospective cohort component. The unit of analysis was the individual, with ecological linkages at the municipal level for geographic analyses. The study integrated nine publicly available data sources from Mexican government agencies, each collected between 2017 and 2023, to examine social security coverage, chronic disease burden, and mortality among PWD. Each data source was analyzed independently using its own complex survey design; no cross-survey individual-level record linkage was attempted, as all datasets are anonymized with non-overlapping sampling frames. A prospective mortality analysis linked baseline disability status from the 2018 wave of the Mexican Health and Aging Study (ENASEM) to vital status at the 2021 follow-up.

### 2.2 Data sources

Nine data sources were used, categorized into individual-level surveys, ecological indicators, and administrative records (Supplementary Tables S1, S2).

#### 2.2.1 Population and housing census 2020

The extended questionnaire (*Cuestionario Ampliado*) of the 2020 Census (INEGI) provided the primary dataset: 15,015,683 person-level records representing 125.5 million expanded population across 32 states (45). Variables included disability functioning (seven domains), social security affiliation, sociodemographics, ethnicity, economic activity, and geographic identifiers (state and municipality codes). Survey design: stratified cluster sampling with strata, primary sampling units (PSUs), and expansion factors.

#### 2.2.2 Multidimensional poverty database (CONEVAL 2022)

CONEVAL's multidimensional poverty measurement database, derived from the ENIGH 2022, provided 309,534 person-level records with pre-calculated binary indicators for six social deprivations and five mutually exclusive poverty categories (46, 47).

#### 2.2.3 National household income and expenditure survey (ENIGH 2022)

The ENIGH 2022 New Series (INEGI) provided 309,684 person-level records across 17 tables, enabling analysis of health expenditure, employment benefits, labor informality, and catastrophic spending at the household level (48).

#### 2.2.4 National survey on discrimination (ENADIS 2022)

The ENADIS 2022 (INEGI) disability module provided 5,696 records from a pre-selected PWD sample, capturing access barriers, rights denial, and discrimination experiences (49).

#### 2.2.5 National employment and social security survey (ENESS 2017)

The ENESS 2017 (INEGI) provided 259,949 individual records with institutional affiliation, pension receipt, active contribution status, and reasons for non-affiliation (50).

#### 2.2.6 National health and nutrition survey (ENSANUT 2022)

The ENSANUT Continua 2022 (INSP) adults questionnaire provided 11,913 records for individuals aged 20+ years (51). Morbidity data included physician-diagnosed chronic conditions, depression screening (Center for Epidemiologic Studies Depression Scale [CES-D], 7-item).

#### 2.2.7 Mexican health and aging study (ENASEM/MHAS 2018–2021)

ENASEM is a nationally representative longitudinal study of adults aged 50+ (52). The 2021 wave provided 15,739 living respondents with health outcomes, and the master follow-up file tracked 18,219 individuals for inter-wave mortality (1,614 deaths). The 2018 wave constructed variables (17,114 records) provided baseline disability status, education, chronic conditions, and social security affiliation for prospective mortality analysis.

#### 2.2.8 Ecological and administrative sources

Municipal marginalization indices (CONAPO 2020; 2,469 municipalities) (53) and health infrastructure data (DGIS 2023; 21,624 facilities across 12 institutions) (54) were merged ecologically with Census data via harmonized 5-digit municipal codes (ENT+MUN). Match rate: 100% of Census person-records.

### 2.3 Variable definitions

#### 2.3.1 Disability (exposure)

Disability was operationalized using source-specific measures, including the Washington Group (WG) methodology (6) where available (Supplementary Table S1). In the Census 2020 (45), six functional domains were assessed on a 4-point scale (1 = no difficulty to 4 = cannot do it); disability was defined as scoring ≥3 in any domain or reporting a mental health condition. In the ENSANUT 2022 (51), the WG Short Set was applied with aided versions for vision and hearing when assistive devices were used. In the ENASEM (52), disability was defined as any basic activity of daily living (ADL; N_ABVD≥1) or instrumental ADL (IADL; N_AIVD≥1) limitation, with a 4-level severity classification: no disability, mild (IADL only), moderate (1–2 ADL), and severe (3+ ADL). The ENIGH (48) uses an inverted scale (1 = cannot do it, 2 = great difficulty, 3 = some difficulty, 4 = no difficulty); disability was defined as any domain ≤ 2 (“great difficulty or cannot do it”), a threshold conceptually aligned with the Census Washington Group criterion of ≥3 (“a lot of difficulty or worse”) rather than with the broader “any difficulty” definition. The CONEVAL database (46) provides a pre-calculated binary indicator. In the ENESS 2017 (50), disability status was classified as limitation (AFI_DISCA = 1) or disability (AFI_DISCA = 2); both categories were combined as PWD. Scale direction was verified empirically against the observed response distribution and cross-database prevalence checks; the chosen ≤ 2 threshold yielded a survey-weighted disability prevalence consistent with the Census Washington Group estimate, whereas the broader “any difficulty” ( ≤ 3) definition matched the national disability-or-limitation figure (Supplementary Table S11). To confirm that the catastrophic-expenditure findings were not driven by an overly inclusive disability definition, we conducted a sensitivity analysis re-estimating the models under a strict ENIGH threshold (“cannot do it” only; Supplementary Table S11).

#### 2.3.2 Outcomes

The primary outcome was lack of social security affiliation, defined as reporting no health service institution (DHSERSAL1 = “09” in the Census). Secondary outcomes included: type of social security (contributory, non-contributory, private, unaffiliated); six CONEVAL social deprivation indicators; catastrophic health expenditure (WHO definition: out-of-pocket spending >40% of capacity to pay (18); alternative: >10% of income); physician-diagnosed chronic conditions (ENSANUT: diabetes, hypertension, cardiovascular disease, chronic kidney disease, depression, high cholesterol, multimorbidity [≥2 conditions]); depressive symptomatology (CES-D 7-item, threshold ≥9); hospitalization in the past 12 months; and inter-wave mortality (ENASEM: died between 2018 and 2021 waves).

#### 2.3.3 Covariates

Covariates varied by analysis. Census logistic models included sex (male/female), age group (15–29 [reference], 30–44, 45–59, 60–74, 75+), education (no schooling [reference], primary, lower secondary, upper secondary, higher education), indigenous self-identification, indigenous language, and rural residence (< 2,500 inhabitants). Education was derived from accumulated schooling years (ESCOACUM, converted to integer to avoid a known leading-zero coding issue). ENSANUT models additionally adjusted for education level and indigenous language. ENASEM models adjusted for education, social security type, and chronic condition count. Intersectional analyses used a 2 × 2 × 2 factorial design crossing disability, indigenous identity, and female sex.

### 2.4 Statistical analysis

#### 2.4.1 Survey design

All nationally representative surveys employ complex, multi-stage sampling designs that must be accounted for to produce valid point estimates and standard errors. For each data source, the analysis incorporated three design elements: the primary sampling unit (PSU, which captures clustering), the stratification variable (which captures geographic and socioeconomic stratification), and the expansion factor (which projects sample observations to population-level estimates). Variance estimation used Taylor series linearization as implemented in the R survey package (55, 56). When a stratum contained only a single PSU, the “adjust” method was applied, which centers the stratum at the grand mean to avoid undefined variance estimates.

#### 2.4.2 Descriptive analysis

Survey-weighted prevalence estimates with 95% confidence intervals were computed for social security coverage, chronic disease prevalence, and social deprivations, stratified by disability status. Differences in proportions were tested on the full weighted samples using the Rao–Scott (survey-adjusted Pearson) χ^2^ test. For continuous variables in the Census (age, years of schooling), between-group differences were assessed with the Wilcoxon rank-sum test computed on a random subsample of 50,000 records; rank-based tests on the full 15-million-record file are computationally prohibitive and a subsample of this size yields stable, essentially identical inference given the very large effective sample. Age-stratified comparisons were conducted to assess potential confounding by age structure, given the known susceptibility of aggregate disability–coverage associations to amalgamation effects of the type described by Simpson's paradox (57).

#### 2.4.3 Regression modeling

Five nested survey-weighted logistic regression models examined the association between disability and SS exclusion among Census 2020 (45) respondents aged ≥15 (N ≈ 10.7 million):


$$\begin{array}{l} \text{logit}\left(P\left[\text{no SS}\right]\right)=\beta_{0}+\beta_{1}\cdot \text{disability}+\beta \cdot \text{X} \end{array}$$ (1)

where **X** expanded progressively: M1 (unadjusted), M2 (+sex, age group), M3 (+education), M4 (+indigenous, rural), and M5 (disability × sex + disability × indigenous interactions). Covariates were selected a priori as established social determinants of social protection coverage and entered in this pre-specified progressive sequence to render the contribution of each confounding domain transparent, rather than through any data-driven variable-selection procedure. The two interaction terms (disability × sex and disability × indigenous identity) were likewise specified a priori to test the study's intersectional hypothesis and were not selected **post hoc**. In logistic regression, interaction terms operate multiplicatively on the odds ratio scale: the net OR for a subgroup defined by multiple characteristics is obtained by multiplying the main effect OR by each applicable interaction OR. For example, the net OR for an indigenous woman with disability equals OR_disc_×OR_disc × female_×OR_disc × indigenous_. An interaction OR above 1.0 therefore does not indicate increased risk in isolation; rather, it indicates that the combined association exceeds what would be predicted from the individual terms alone (a supermultiplicative effect on the odds-ratio scale). Results are reported as odds ratios (OR) with 95% confidence intervals (CI).

For chronic disease prevalence (ENSANUT (51), 9 conditions) and health outcomes (ENASEM (52), 7 outcomes), two survey-weighted logistic models were fitted per outcome: Model A (age + sex) and Model B (fully adjusted: + education + indigenous language [ENSANUT] or + education + SS type [ENASEM]). Benjamini–Hochberg (BH) false discovery rate correction (58) was applied across simultaneous comparisons within each source.

Five nested logistic models estimated prospective three-year mortality (ENASEM 2018 → 2021 (52), *N* = 10,268 linked individuals):


$$\begin{array}{l} \text{logit}\left(P\left[{\text{died}}_{2021}\right]\right)=\beta_{0}+\beta_{1}\cdot {\text{disability}}_{2018}+\beta \cdot {\text{X}}_{2018} \end{array}$$ (2)

progressing from disability only (M1) through full adjustment for age, sex, education, SS type, and baseline chronic conditions (M4). A severity-gradient model (M5) replaced the binary disability term with the 4-level severity classification. Because the observed three-year mortality among PWD was high—a level at which odds ratios materially overestimate risk ratios—we additionally estimated risk ratios for the mortality outcome using modified Poisson regression with robust (sandwich) standard errors and, as a cross-check, marginal standardization (g-computation) over the covariate distribution of the analytic sample (Supplementary Table S10). These prospective mortality models were fitted as unweighted binomial cohort models on the linked ENASEM analytic sample; the resulting estimates should therefore be interpreted as within-cohort associational estimates rather than design-based population mortality rates.

#### 2.4.4 Intersectional analysis

An additive decomposition framework (20, 21) examined compounded SS exclusion across eight intersectional profiles (disability × indigenous × female). Super-additive effects were identified when observed exclusion rates exceeded the sum of individual penalty terms relative to the reference group (non-indigenous male without disability). This decomposition is performed on the absolute (percentage-point) risk scale, on which an excess over the sum of individual penalties is termed *super-additive*; the corresponding interaction terms estimated in the multiplicative logistic model (M5; odds ratios above 1.0) instead capture departure from multiplicativity on the odds scale, i.e., a *supermultiplicative* effect. We report both and label each result by the scale to which it refers.

#### 2.4.5 Geographic analysis

Healthcare infrastructure was classified at the municipal level using DGIS 2023 (54) sectorial data (21,624 facilities across 12 health-sector institutions). Municipalities were categorized into three tiers based on hospital bed availability: *desert* (zero hospital beds), *limited* (1–30 beds, below the national median among municipalities with hospitals), and *adequate* (>30 beds). This classification operationalizes the concept of healthcare (“medical”) deserts—areas with little or no access to care (59)—for Mexican municipalities using inpatient (hospital-bed) capacity. Because no standard bed-based threshold has been established for Mexico, the “limited” cutoff was set empirically at the national median number of hospital beds among municipalities with at least one hospital. Absolute inpatient capacity was used as the classification axis because the *desert* tier (zero beds) reflects a denominator-independent structural absence of inpatient care, and because municipal population denominators are subject to boundary changes and inter-censal estimation volatility; the resulting tiers were nonetheless strongly concordant with population-adjusted supply, with mean hospital-bed density rising monotonically across the desert, limited, and adequate tiers (0, 0.63, and 1.26 beds per 1,000 population, respectively). Triple vulnerability was defined as the intersection of disability, SS exclusion, and residence in a municipality classified as desert or limited. Municipality-level disability prevalence rates were computed from Census data and linked to hospital bed density and CONAPO (53) marginalization indices.

### 2.5 Software

All statistical analyses were performed in R version 4.5.3. Key packages included: survey 4.5 and srvyr 1.3.1 for complex survey design estimation (55); broom 1.0.12 for tidy model output; nnet 7.3–20 for multinomial regression; sandwich 3.1.1 and lmtest 0.9–40 for risk ratios and robust (sandwich) variance estimation; EValue 4.1.4 for selection-bias E-values; dplyr 1.2.1 and readr 2.2.0 (within tidyverse 2.0.0) for data management; and ggplot2 4.0.2, patchwork 1.3.2, scales 1.4.0, viridis 0.6.5, and ggrepel 0.9.8 for visualization. Data extraction utilities and the variable dictionary were generated using Python 3.12 with the openpyxl 3.1.2 package. Analysis scripts are deposited in Zenodo (see Data Availability Statement).

### 2.6 Ethical statement

Ethical review and approval were waived for this study. This research used exclusively publicly available, de-identified microdata from official open government sources (INEGI, CONEVAL, INSP, CONAPO, and Secretaría de Salud). No individual-level identifiable information was accessed or used. In accordance with Article 17 of Mexico's *Reglamento de la Ley General de Salud en Materia de Investigación para la Salud* (60) and institutional guidelines, ethics committee approval was not required for analyses based on open administrative and survey data. Informed consent was not required because this study exclusively used anonymized secondary data from national surveys and publicly available open-access databases. No direct contact was made with participants, and no attempts were made to re-identify individuals.

## 3 Results

### 3.1 Study population characteristics

**Figure 1:**
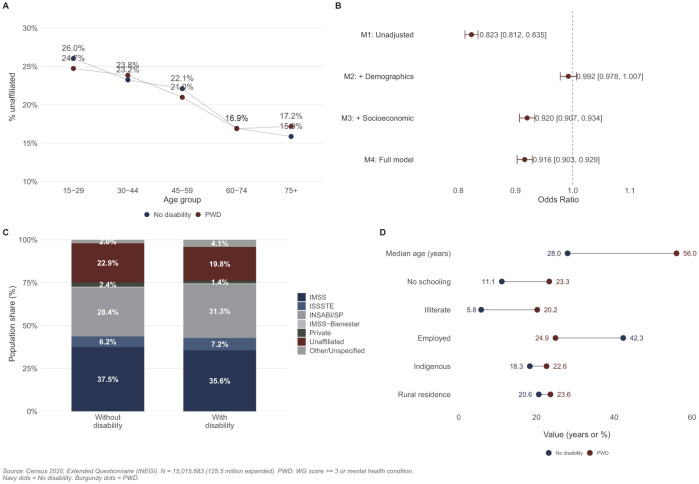
Social security coverage disparities by disability status and age-structure confounding. **(A)** Age-stratified proportion of unaffiliated population by disability status, showing that the aggregate coverage advantage is driven by the older age structure of the PWD population rather than consistent lower exclusion across all working ages. **(B)** Forest plot of nested logistic regression models (M1–M4) for lack of SS affiliation; the unadjusted protective effect (OR = 0.82) disappears after demographic adjustment (OR = 0.99). **(C)** Social security institutional composition by disability status, showing the structural shift from contributory (IMSS/ISSSTE) to non-contributory (INSABI) coverage among PWD. An “Other/Unspecified” segment (small administrative categories) is included so that both bars sum to 100%; IMSS-Bienestar (0.6% in both groups) is shown in the legend but not separately labeled owing to its small segment size. **(D)** Dumbbell plot of key sociodemographic gaps between PWD and non-disabled population: median age (56 vs. 28 years), no schooling (23.3% vs. 11.1%), illiteracy (20.2% vs. 5.8%), and employment rate (24.9% vs. 42.3%). Source: Census 2020 (INEGI). *N* = 15,015,683 (125.5 million expanded). PWD, persons with disabilities; SS, social security; OR, odds ratio; WG, Washington Group; IMSS, Instituto Mexicano del Seguro Social; ISSSTE, Instituto de Seguridad y Servicios Sociales de los Trabajadores del Estado; INSABI, Instituto de Salud para el Bienestar.

The 2020 Census (45) extended questionnaire yielded 15,015,683 person-level records representing an expanded population of 125.5 million individuals. Of these, 6,821,263 (5.4%) met the Washington Group criteria for disability. Compared to the non-disabled population, PWD were substantially older (median age 56 vs. 28 years; 45.9% aged ≥60 vs. 10.2%), more likely to be female (52.4% vs. 51.2%), and showed marked socioeconomic disadvantage: lower median education (6 vs. 9 years of schooling), three-fold higher illiteracy (20.2% vs. 5.8%), half the employment rate (24.9% vs. 42.3%), and 29% lower median income among workers ($4,300 vs. $6,020 MXN/month). PWD were more likely to self-identify as indigenous (22.6% vs. 18.3%) and to reside in rural areas (23.6% vs. 20.6%). Social security coverage showed an apparently contradictory aggregate pattern: PWD had a lower unaffiliated rate (19.8% vs. 22.9%) but relied disproportionately on non-contributory programs (Instituto de Salud para el Bienestar [INSABI] 31.3% vs. 28.4%) while having half the rate of private insurance (1.4% vs. 2.4%). Full demographic, socioeconomic, and social security characteristics are presented in Table 1, and the key gaps are visualized in Figure 1D.

**Table 1:** Demographic, socioeconomic, and social security characteristics by disability status, Population and Housing Census 2020, Mexico (weighted estimates).

	Total	Without disability	With disability	
Characteristic	(*N* = 125,510,021)	(*N* = 118,688,758)	(*N* = 6,821,263)	*p*
*Demographics*				
Age, years, median (IQR)	29 (14–47)	28 (14–45)	56 (31–73)	< 0.001^*a*^
Female sex, No. (%)	64,369,668 (51.3)	60,792,468 (51.2)	3,577,200 (52.4)	< 0.001^*b*^
Age group, No. (%)				< 0.001^*b*^
0–14	31,938,165 (25.4)	31,097,854 (26.2)	840,311 (12.3)	
15–29	31,093,621 (24.8)	30,304,738 (25.5)	788,883 (11.6)	
30–44	26,658,964 (21.2)	25,879,653 (21.8)	779,311 (11.4)	
45–59	20,615,945 (16.4)	19,331,978 (16.3)	1,283,967 (18.8)	
60–74	11,134,908 (8.9)	9,519,476 (8.0)	1,615,432 (23.7)	
≥75	4,068,418 (3.2)	2,555,059 (2.2)	1,513,359 (22.2)	
*Socioeconomic*				
Education, years, median (IQR)	9 (5–12)	9 (5–12)	6 (1–9)	< 0.001^*a*^
Education level, No. (%)				< 0.001^*b*^
No schooling	14,020,535 (11.8)	12,514,877 (11.1)	1,505,658 (23.3)	
Primary (1–6)	33,247,486 (27.9)	30,617,050 (27.2)	2,630,436 (40.7)	
Lower secondary (7–9)	29,709,837 (24.9)	28,557,160 (25.3)	1,152,677 (17.8)	
Upper secondary (10–12)	22,659,700 (19.0)	22,011,711 (19.5)	647,989 (10.0)	
Higher education (≥13)	19,590,353 (16.4)	19,063,181 (16.9)	527,172 (8.2)	
Illiterate, No. (%)	8,314,979 (6.6)	6,939,319 (5.8)	1,375,660 (20.2)	< 0.001^*b*^
Employed, No. (%)	51,847,115 (41.3)	50,151,098 (42.3)	1,696,017 (24.9)	< 0.001^*b*^
Retired/pensioner, No. (%)	14,549,407 (11.6)	14,258,363 (12.0)	291,044 (4.3)	< 0.001^*b*^
Monthly income, MXN, median (IQR)^*c*^	6,020 (4,000–9,000)	6,020 (4,300–9,200)	4,300 (2,580–7,000)	< 0.001^*a*^
*Ethnicity and residence*				
Indigenous self-identification, No. (%)	23,229,089 (18.5)	21,690,625 (18.3)	1,538,464 (22.6)	< 0.001^*b*^
Indigenous language speaker, No. (%)	7,522,496 (6.0)	7,007,560 (5.9)	514,936 (7.5)	< 0.001^*b*^
Afrodescendant, No. (%)	2,481,967 (2.0)	2,330,919 (2.0)	151,048 (2.2)	< 0.001^*b*^
Rural residence (< 2,500 inhab.), No. (%)	26,028,675 (20.7)	24,420,066 (20.6)	1,608,609 (23.6)	< 0.001^*b*^
*Social security coverage*				
Unaffiliated, No. (%)	28,579,919 (22.8)	27,228,024 (22.9)	1,351,895 (19.8)	< 0.001^*b*^
IMSS, No. (%)	46,967,761 (37.4)	44,536,253 (37.5)	2,431,508 (35.6)	< 0.001^*b*^
ISSSTE (federal + state), No. (%)	7,846,203 (6.3)	7,353,090 (6.2)	493,113 (7.2)	< 0.001^*b*^
INSABI/Seguro Popular, No. (%)	35,833,246 (28.6)	33,699,093 (28.4)	2,134,153 (31.3)	< 0.001^*b*^
IMSS-Bienestar, No. (%)	708,617 (0.6)	666,624 (0.6)	41,993 (0.6)	< 0.001^*b*^
Private insurance, No. (%)	2,954,154 (2.4)	2,860,795 (2.4)	93,359 (1.4)	< 0.001^*b*^

^*a*^Wilcoxon rank-sum test on a random sub-sample of *n* = 50,000 (see Methods, Section 2.4.2). ^*b*^Pearson's χ^2^ test (weighted). ^*c*^Among workers with reported income (*n* = 48,223,548 weighted). All estimates weighted by Census expansion factor.

Social security categories are mutually exclusive; small residual categories (Pemex/Defense/Navy, other institution, and not specified) are not shown, so the listed categories may not sum to 100% (95.9% for PWD; 98.0% without disability). PWD, persons with disabilities; SS, social security; IQR, interquartile range; IMSS, Instituto Mexicano del Seguro Social; ISSSTE, Instituto de Seguridad y Servicios Sociales de los Trabajadores del Estado; INSABI, Instituto de Salud para el Bienestar.

### 3.2 Social security coverage and economic consequences

In the unadjusted comparison, PWD appeared better covered than the general population, with a lower proportion unaffiliated (19.8% vs. 22.9%). However, 46% of PWD are aged 60 or older (compared to 10% of the general population), and older adults have near-universal coverage through non-contributory programs. Age-stratified analysis revealed that the apparent aggregate advantage was attributable to non-contributory programs at older ages rather than employment-linked coverage, and that PWD coverage was disproportionately non-contributory (31.3% vs. 28.4% in INSABI/Seguro Popular; *p* < 0.001) with half the rate of private insurance (1.4% vs. 2.4%; *p* < 0.001; Figure 1C).

Five nested survey-weighted logistic regression models quantified this pattern (Table 2; Figure 1B). The unadjusted model yielded an OR of 0.82 (95% CI: 0.81–0.83), suggesting PWD were less likely to lack SS. After adjusting for age and sex (M2), the association became null (OR = 0.99; 95% CI: 0.98–1.01; *p* = 0.30). The full model (M4), additionally adjusting for education, indigenous identity, and rural residence, yielded OR = 0.92 (95% CI: 0.90–0.93; *p* < 0.001), indicating a modest residual protective effect. The decisive change for the disability association occurs at M2: adjusting for age and sex alone moves the disability odds ratio from 0.82 to 0.99, so that the apparent aggregate advantage is essentially fully explained by the older age structure of PWD, with subsequent covariates producing only minor further change. Overall model fit improved progressively across the nested specifications (McFadden pseudo-*R*^2^ rising from 0.0004 to 0.014; residual deviance in Table 2 and Supplementary Table S13). The small residual protective association in M4 should not be read as genuine inclusion: as shown below, it is concentrated in non-contributory programs that confer nominal rather than effective protection, and it is eliminated for multiply marginalized subgroups. This finding was robust to alternative disability thresholds: the M4 OR ranged from 0.86 (strict: “cannot do it” only) to 0.92 (broad: “some difficulty” or worse) across a 13-fold range of disability prevalence (Supplementary Table S3). An interaction model (M5) showed that the apparent protective association of disability (main effect OR = 0.87; 95% CI: 0.85–0.88) was attenuated for women (disc × female interaction OR = 1.07; 95% CI: 1.04–1.09; *p* < 0.001) and indigenous persons (disc × indigenous interaction OR = 1.10; 95% CI: 1.07–1.13; *p* < 0.001; Table 2). Because these interaction ORs are multiplicative modifiers of the main disability effect, the net disability odds ratio for an indigenous woman—that is, the association of disability with exclusion *conditional on* being an indigenous woman, not the absolute exclusion odds of that profile relative to the reference group—is approximately 0.87 × 1.07 × 1.10 ≈ 1.02, indicating that the residual protective effect of disability observed in M4 is effectively erased for the most multiply marginalized profile.

**Table 2:** Nested survey-weighted logistic regression models for lack of social security affiliation among adults aged ≥15, Census 2020.

Model	Covariates	N	OR	95% CI	*p*	Deviance
M1	Disability (unadjusted)	10,739,396	0.82	0.81–0.83	< 0.001	11,539,454
M2	+ Sex, age group	10,739,396	0.99	0.98–1.01	0.298	11,466,449
M3	+ Education	10,717,409	0.92	0.91–0.93	< 0.001	11,359,142
M4	+ Indigenous, rural	10,717,409	0.92	0.90–0.93	< 0.001	11,348,643
M5	+ disc × sex, disc × indigenous	10,717,409	0.87	0.85–0.88	< 0.001	11,348,362
	disc × female interaction		1.07	1.04–1.09	< 0.001	
	disc × indigenous interaction		1.10	1.07–1.13	< 0.001	

M = nested multivariate logistic regression model. OR = disability odds ratio (main effect in M1–M4; interaction terms for M5).

Quasibinomial family; deviance = residual deviance. N decreases from M2 to M3 due to 21,987 records (0.2%) with missing education data.

Full coefficients for all models in Supplementary Table S9.

OR, odds ratio; CI, confidence interval; SS, social security; PWD, persons with disabilities.

The institutional composition of SS coverage further revealed that the apparent protection of PWD reflects a structural shift from contributory to non-contributory coverage rather than genuine inclusion (Figure 1C). While Instituto Mexicano del Seguro Social (IMSS) coverage was comparable (35.6% vs. 37.5%), PWD were more likely to be enrolled in INSABI/Seguro Popular (31.3% vs. 28.4%; *p* < 0.001) and less likely to hold private insurance (1.4% vs. 2.4%; *p* < 0.001), consistent with lower rates of formal employment (24.9% vs. 42.3%; Table 1). Among unaffiliated PWD (ENESS 2017 (50); *n* = 2,351), the most frequently reported reasons for non-affiliation were lack of formal employment (44.7%; 95% CI: 41.4–48.0) and unawareness of enrollment requirements (44.2%; 40.9–47.6), followed by absence of nearby medical facilities (17.1%) and costly procedures (14.1%), suggesting that labor market exclusion and information barriers are among the primary structural factors underlying the coverage gap. Only 9.1% of PWD were actively contributing to social security, compared to 23.3% of the non-disabled population (Supplementary Table S4). Because the ENESS predates the 2020 restructuring of non-contributory coverage, these specific reasons for non-affiliation characterize the late–Seguro Popular period and may not fully reflect the current INSABI/IMSS-Bienestar configuration.

The economic disparities associated with this coverage gap were substantial (Table 3). Households with PWD members had higher median quarterly health expenditure ($293 vs. $147 MXN; *p* < 0.001), spending a larger share of income on health (3.6% vs. 2.1%; *p* < 0.001). Catastrophic health expenditure, defined as out-of-pocket spending exceeding 40% of capacity to pay, was 2.3 times more prevalent among PWD households (2.8% vs. 1.2%; 95% CI: 2.5–3.2 vs. 1.1–1.3; *p* < 0.001). By the alternative threshold (>10% of income), the rate was 8.1% vs. 3.5% (*p* < 0.001), with the sharpest disparity in the poorest quintile (11.6% vs. 5.7%; *p* < 0.001). Among working-age adults (18–59), retirement savings (Administradoras de Fondos para el Retiro [AFORE]) coverage was consistently lower among PWD (overall *p* < 0.001), and the gap widened progressively with age: 4.9pp at ages 18–29 (*p*=0.08), 7.3pp at ages 30–44 (*p* < 0.001), and 10.6pp at ages 45–59 (*p* < 0.001; Table 3). Among older adults (≥65), PWD were less likely to receive a contributory pension (28.4% vs. 35.3%; *p* < 0.001) and more dependent on the non-contributory Programa de Adultos Mayores (37.0% vs. 29.8%; *p* < 0.001).

**Table 3:** Economic consequences of disability: health expenditure, catastrophic spending, and pension coverage (ENIGH 2022, CONEVAL 2022).

Indicator	Without PWD	With PWD	*p*
*Health expenditure (household-level, ENIGH 2022)*			
Median quarterly health spending, MXN	147	293	< 0.001
Health spending as % of income, mean	2.1 (1.9–2.3)	3.6 (3.4–3.8)	< 0.001
Catastrophic (WHO: >40% capacity to pay), %	1.2 (1.1–1.3)	2.8 (2.5–3.2)	< 0.001
Catastrophic (>10% of income), %	3.5 (3.3–3.7)	8.1 (7.5–8.6)	< 0.001
*Catastrophic spending by income quintile (>10% threshold)*			
Q1 (poorest)	5.7 (5.1–6.2)	11.6 (10.4–12.8)	< 0.001
Q3 (middle)	2.9 (2.5–3.3)	6.2 (5.1–7.3)	< 0.001
Q5 (wealthiest)	3.3 (2.9–3.8)	7.8 (6.4–9.2)	< 0.001
*Retirement savings—AFORE coverage (workers 18–59, ENIGH 2022)*			
Ages 18–29, %	28.9 (28.1–29.6)	24.0 (18.9–29.0)	0.08
Ages 30–44, %	33.3 (32.6–34.1)	26.0 (22.5–29.6)	< 0.001
Ages 45–59, %	30.6 (29.8–31.4)	20.0 (17.7–22.4)	< 0.001
*Pension source (adults ≥65, CONEVAL 2022)*			
Contributory retirement pension, %	35.3 (34.4–36.3)	28.4 (27.0–29.8)	< 0.001
Non-contributory PAM, %	29.8 (28.8–30.8)	37.0 (35.5–38.6)	< 0.001
No pension, %	34.9 (33.8–35.9)	34.6 (33.0–36.2)	0.73

95% CI in parentheses. Catastrophic expenditure: WHO definition and alternative threshold.

AFORE, Administradoras de Fondos para el Retiro; PAM, Programa de Adultos Mayores.

*p*-values: survey-weighted χ^2^ (proportions) or Wilcoxon rank-sum (medians).

PWD, persons with disabilities; CI, confidence interval; SS, social security; pp, percentage points.

Survey-weighted logistic regression indicated that disability was independently associated with catastrophic health expenditure after adjusting for income quintile, household size, presence of older adult members, and rural residence (Table 4). Using the WHO definition (>40% of capacity to pay), households with PWD members had 2.02 times the odds of catastrophic spending (95% CI: 1.73–2.35; *p* < 0.001). By the alternative threshold (>10% of income), the fully adjusted OR was 1.96 (95% CI: 1.78–2.16; *p* < 0.001). The attenuation from unadjusted (OR ≈ 2.4) to fully adjusted (OR ≈ 2.0) was modest, indicating that the association between disability and catastrophic expenditure is largely independent of income level and household composition (full model coefficients in Supplementary Table S5). This association was not an artifact of the ENIGH disability definition: under a strict threshold (“cannot do it” only; household disability prevalence reduced roughly five-fold), the fully adjusted odds ratio was, if anything, larger (WHO definition OR = 2.44, 95% CI: 1.89–3.15; alternative definition OR = 2.27, 95% CI: 1.95–2.64; Supplementary Table S11).

**Table 4:** Logistic regression models for catastrophic health expenditure by household disability status, ENIGH 2022.

Definition	Model	OR	95% CI	*p*
*WHO definition (>40% capacity to pay)*				
	M1: Unadjusted	2.39	2.07–2.76	< 0.001
	M2: + Income quintile, HH size	2.13	1.84–2.46	< 0.001
	M3: + Older adult member, rural	2.02	1.73–2.35	< 0.001
*Alternative definition (>10% income)*				
	M1: Unadjusted	2.41	2.20–2.64	< 0.001
	M2: + Income quintile, HH size	2.35	2.14–2.57	< 0.001
	M3: + Older adult member, rural	1.96	1.78–2.16	< 0.001

M = nested multivariate logistic regression model. OR, odds ratio for PWD household.

Survey-weighted (quasibinomial). *N* = 90,102 households. Full M3 coefficients in Supplementary Table S5.

OR, odds ratio; CI, confidence interval; PWD, persons with disabilities; HH, household.

### 3.3 Intersectional exclusion

**Figure 2:**
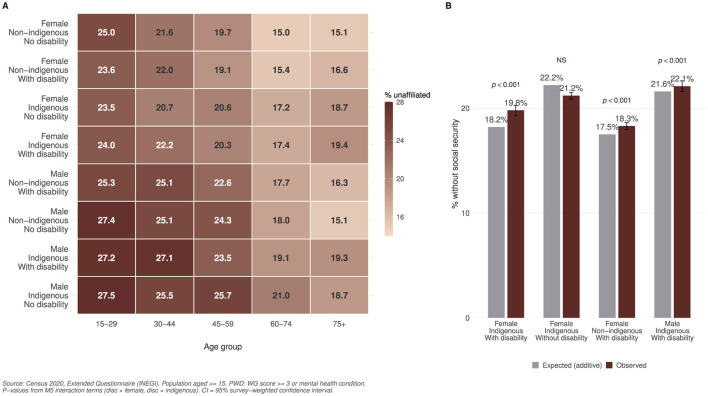
Intersectional social security exclusion by disability, indigenous identity, and sex. **(A)** Heatmap of SS exclusion rates (% unaffiliated) across eight intersectional profiles and five age groups, showing widening gaps during working age. **(B)** Observed vs. expected (additive) exclusion rates for multiply marginalized profiles with 95% CI on observed rates. Super-additive excess and *p*-values from M5 interaction terms annotated below each profile. Indigenous women with disability show +1.6 percentage points above the expected additive penalty (*p* < 0.001). Source: Census 2020 (INEGI). Population aged ≥ 15. *N* = 10,717,409. SS, social security; CI, confidence interval; pp, percentage points; PWD, persons with disabilities.

Analysis of eight intersectional profiles (disability × indigenous identity × female sex) revealed super-additive effects on SS exclusion among multiply marginalized groups (Figure 2; Table 5). Using non-indigenous males without disability as the reference group, additive decomposition showed that indigenous women with disability experienced an exclusion rate 1.6 percentage points higher than predicted by the sum of individual penalties (observed 19.8% vs. expected 18.2%; 95% CI for observed: 19.3–20.3; *p* < 0.001), non-indigenous women with disability showed a 0.9pp excess (observed 18.3%; 95% CI: 18.0–18.6; *p* < 0.001), and indigenous men with disability a 0.6pp excess (observed 22.1%; 95% CI: 21.6–22.6; *p* < 0.001; Figure 2B; Table 5).

**Table 5:** Intersectional effects on social security exclusion: super-additive decomposition and M5 interaction terms, Census 2020.

Profile	Obs. (%)	95% CI	Exp. (%)	Excess (pp)	Effect	Interaction term
Female, indigenous, PWD	19.8	19.3–20.3	18.2	+1.6	Super-additive	disc × fem + disc × indig
Female, non-Indig., PWD	18.3	18.0–18.6	17.5	+0.9	Super-additive	disc × fem: 1.07^***^
Male, indigenous, PWD	22.1	21.6–22.6	21.6	+0.6	Super-additive	disc × indig: 1.10^***^
Female, Indig., no disab.	21.2	20.9–21.5	22.2	−1.0	Sub-additive	—
*M5 interaction terms (all *p* < 0.001)*						
disc × female	OR = 1.07 (1.04–1.09)		Disability compounds female exclusion			
disc × indigenous	OR = 1.10 (1.07–1.13)		Disability compounds indigenous exclusion			

Obs./Exp. = observed/expected (additive) exclusion rate. 95% CI = survey-weighted. ^***^p < 0.001.

Reference: non-indigenous male without disability (24.7%). M5: *N*≈10.7M, adjusted for age, education, rural.

OR, odds ratio; CI, confidence interval; SS, social security; PWD, persons with disabilities; pp, percentage points.

These super-additive patterns were corroborated by the interaction terms in Model M5 (Table 5). The interaction ORs above 1.0 (disability × indigenous = 1.10; disability × female = 1.07) do not indicate increased risk in isolation; rather, they are multiplicative modifiers of the main disability effect (OR = 0.87). In practice, each interaction OR erodes the apparent protective association of disability observed within the reference stratum (non-indigenous males): the net disability OR (disabled vs. non-disabled within the same stratum) is approximately 0.87 × 1.07=0.93 for a non-indigenous woman (modest residual protection), 0.87 × 1.10=0.96 for an indigenous man (near null), and 0.87 × 1.07 × 1.10 ≈ 1.02 for an indigenous woman (protection eliminated). In other words, disability is associated with exclusion levels that exceed what would be expected from the sum of individual vulnerabilities alone, and for the most marginalized profiles the apparent aggregate protection disappears entirely. The age-stratified heatmap (Figure 2A) further showed that the intersectional gap between the most and least vulnerable profiles widened during working age (4.0pp at ages 15–29 to 6.6pp at ages 45–59) before narrowing at 60+, consistent with the equalizing role of non-contributory coverage.

### 3.4 Chronic disease prevalence and healthcare utilization

#### 3.4.1 Morbidity burden

All nine conditions assessed in the ENSANUT 2022 (51) (*N* = 11,913 adults aged 20+; 1,309 PWD) were significantly more prevalent among PWD after Benjamini–Hochberg correction (Table 6; Supplementary Figures S1, S2). All ORs reported below are fully adjusted for age, sex, education, and indigenous language. The largest disparities were observed for depressive symptomatology (CES-D screen: 84.1% vs. 61.9%; OR = 2.92; 95% CI: 2.43–3.51), chronic kidney disease (4.3% vs. 1.3%; OR = 2.57; 1.63–4.04), multimorbidity (40.1% vs. 14.4%; OR = 2.25; 1.89–2.68), and physician-diagnosed depression (22.6% vs. 10.4%; OR = 2.23; 1.80–2.75). Adding socioeconomic covariates to the age-sex base model attenuated ORs by less than 5% for all conditions (Supplementary Figure S2), suggesting that these associations are not substantially confounded by socioeconomic position.

**Table 6:** Disease prevalence and fully adjusted odds ratios by disability status, ENSANUT 2022 (BH-corrected).

	No disability	PWD			
Condition	(*n* = 10,604)	(*n* = 1,309)	OR_adj_	95% CI	*p*_adj_
Depression (CES-D screen)	6,596 (61.9)	1,090 (84.1)	2.92	2.43–3.51	< 0.001
CKD	139 (1.3)	55 (4.3)	2.57	1.63–4.04	< 0.001
Multimorbidity (≥2)	1,557 (14.4)	524 (40.1)	2.25	1.89–2.68	< 0.001
Accident (12 months)	603 (5.8)	164 (12.2)	2.27	1.75–2.95	< 0.001
Depression (diagnosed)	1,157 (10.4)	285 (22.6)	2.23	1.80–2.75	< 0.001
Any CVD event	434 (4.2)	155 (12.0)	2.10	1.63–2.71	< 0.001
Hypertension	1,733 (15.8)	528 (40.5)	1.87	1.57–2.23	< 0.001
High cholesterol	1,615 (15.4)	370 (27.2)	1.47	1.22–1.78	< 0.001
Diabetes	1,186 (10.8)	358 (25.7)	1.46	1.20–1.76	< 0.001

Values are *n* (survey-weighted %). OR_adj_ = fully adjusted OR (age + sex + education + indigenous language).

*p*_adj_ = BH-corrected. Survey-weighted logistic regression. N = 11,913 adults aged 20+. ENSANUT 2022.

PWD, persons with disabilities; OR, odds ratio; CI, confidence interval; BH, Benjamini–Hochberg; CES-D, Center for Epidemiologic Studies Depression Scale; CKD, chronic kidney disease; CVD, cardiovascular disease.

Consistent findings in the ENASEM 2021 (52) cohort (*N* = 15,739 adults aged 50+; 3,252 PWD) yielded fully adjusted ORs of 4.12 (95% CI: 2.78–6.11) for stroke, 2.72 (2.26–3.28) for depression, 2.44 (2.02–2.95) for multimorbidity, and 2.31 (1.81–2.95) for hospitalization in the past 12 months (all *p*_adj_ < 0.001 after BH correction; Supplementary Table S6).

#### 3.4.2 Social deprivations

CONEVAL 2022 (46) data confirmed that PWD experienced higher rates of all six social deprivations after adjusting for age and sex (Table 7). The largest adjusted disparities were observed for food insecurity (OR = 2.43; 95% CI: 2.30–2.56; *p* < 0.001), educational lag (OR = 2.32; 2.21–2.43; *p* < 0.001), and poverty (OR = 1.58; 1.50–1.66; *p* < 0.001). Even social security deprivation, using CONEVAL's broader definition that includes employment benefits and pension criteria, remained significantly higher among PWD after adjustment (OR = 1.26; 1.20–1.32; *p* < 0.001). For three indicators—social security, health services, and housing quality deprivation—the crude prevalence was *lower* among PWD than among the non-disabled in unadjusted comparisons (e.g., social security deprivation 41.1% vs. 50.8%), yet the age- and sex-adjusted odds ratio exceeded 1.0. This reversal is a further manifestation of confounding by age: PWD are substantially older (median 62 vs. 30 years), and these deprivations decline steeply with age (e.g., social security deprivation falls from 58% at ages 0–17 to 19% at ages ≥75, reflecting old-age pension coverage). Once age and sex are held constant, the expected higher deprivation burden among PWD emerges, mirroring the coverage paradox observed for affiliation. Among the ENADIS 2022 (49) disability module (*N* = 5,696 PWD), 42.0% reported insufficient preparation at health centers, 13.6% were denied medical care or medications in the past five years, and 16.6% were denied social program benefits (Table 7).

**Table 7:** Social deprivations and access barriers by disability status, CONEVAL 2022 and ENADIS 2022.

	No disability	PWD			
Indicator	(*n* = 287,288)	(*n* = 21,846)	OR_adj_	95% CI	*p*
*Social deprivations (CONEVAL 2022, N = 309,134)*				
Food insecurity	45,277 (15.6)	5,441 (25.1)	2.43	2.30–2.56	< 0.001
Educational lag	53,988 (17.6)	10,363 (44.8)	2.32	2.21–2.43	< 0.001
Poverty (any type)	97,492 (35.9)	8,545 (41.2)	1.58	1.50–1.66	< 0.001
Extreme poverty	18,600 (7.0)	1,667 (8.1)	1.56	1.43–1.71	< 0.001
Basic services deprivation	57,827 (17.6)	5,070 (20.4)	1.36	1.29–1.44	< 0.001
Housing quality deprivation	28,379 (9.1)	1,908 (8.4)	1.36	1.25–1.48	< 0.001
SS deprivation	147,323 (50.8)	9,059 (41.1)	1.26	1.20–1.32	< 0.001
Health services deprivation	107,421 (39.3)	7,610 (36.7)	1.15	1.09–1.20	< 0.001
*Access barriers among PWD (ENADIS 2022, N = 5,696 PWD)*					
	**Weighted % (95% CI)**				
Insufficient preparation at health centers	42.0 (39.7–44.3)				
Denied social program benefits	16.6 (14.9–18.3)				
Denied employment opportunity	16.1 (13.9–18.3)				
Denied medical care or medications	13.6 (12.0–15.1)				
Cost of treatments as main problem	21.7 (20.1–23.4)				

Values are *n* (survey-weighted %). OR_adj_ = adjusted for age and sex (quasibinomial, survey-weighted).

ENADIS: descriptive only (all respondents are PWD by design; no between-group comparison possible).

CONEVAL analytic N = 309,134 after exclusion of 400 records with missing disability status (309,534 total).

For SS, health services, and housing quality deprivation, crude prevalence is lower among PWD yet the age- and sex-adjusted OR exceeds 1.0: a reversal driven by confounding from the older age structure of PWD (these deprivations decline with age; see Section 3.4).

PWD, persons with disabilities; OR, odds ratio; CI, confidence interval; SS, social security.

### 3.5 Prospective 3-year mortality

In the prospective cohort linking ENASEM 2018 (52) baseline (N = 10,268) to 2021 vital status, 960 individuals (9.3%) had disability at baseline (any ADL or IADL limitation). During the 3-year follow-up, 498 deaths occurred, yielding a crude mortality rate of 18.1% among PWD compared to 3.5% among those without disability (*p* < 0.001). A dose–response gradient was evident across disability severity: no disability 3.5%, mild (IADL only) 11.0%, moderate (1–2 ADL) 14.2%, and severe (3+ ADL) 33.3% (*p*_trend_ < 0.001; Supplementary Table S7; Supplementary Figure S3).

**Figure 3:**
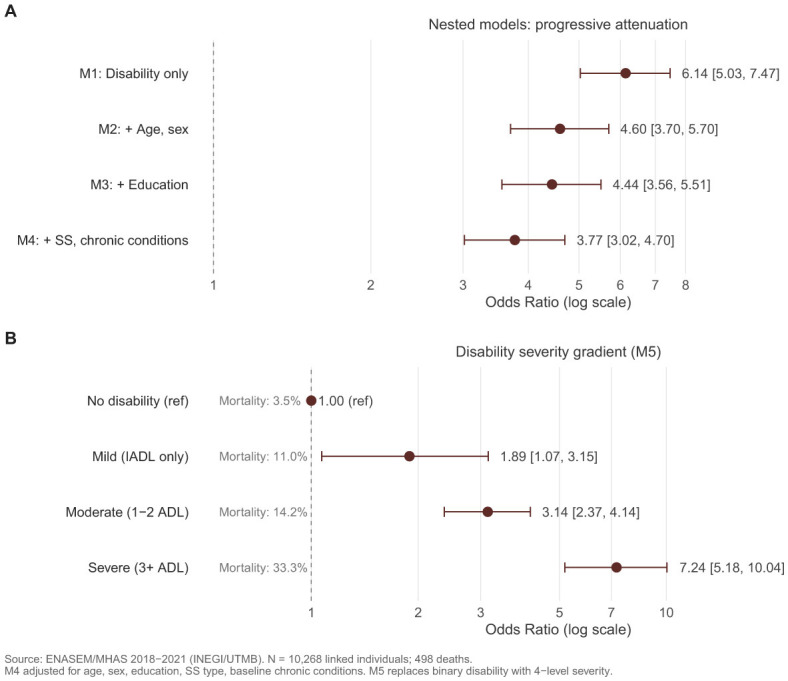
Prospective three-year mortality risk by disability status, ENASEM 2018–2021. **(A)** Nested logistic regression models showing progressive attenuation of the disability–mortality association from unadjusted (OR = 6.14) to fully adjusted (OR = 3.77). **(B)** Disability severity gradient (M5): dose–response relationship from no disability (reference) through mild (OR = 1.89), moderate (OR = 3.14), to severe disability (OR = 7.24). Crude three-year mortality rates annotated for each severity level. Source: ENASEM/MHAS 2018–2021 (INEGI/UTMB). *N* = 10,268 linked individuals; 498 deaths. OR, odds ratio; ADL, activities of daily living; IADL, instrumental ADL.

Five nested logistic regression models quantified the progressive attenuation of the disability–mortality association (Figure 3A; Supplementary Table S14). The unadjusted OR was 6.14 (95% CI: 5.03–7.47). Adjustment for age and sex reduced this to 4.60 (3.70–5.70); adding education yielded 4.44 (3.56–5.51); and the full model, additionally adjusting for SS type and baseline chronic conditions, yielded OR = 3.77 (95% CI: 3.02–4.70; *p* < 0.001). In the severity-gradient model (M5), ORs ranged from 1.89 (1.07–3.15; *p*=0.020) for mild disability to 3.14 (2.37–4.14; *p* < 0.001) for moderate and 7.24 (5.18–10.0; *p* < 0.001) for severe disability (Figure 3B; Supplementary Table S14). The progressive attenuation across nested models is shown in Figure 3A. Because the 3-year mortality rate among PWD was high (18.1%), at which the odds ratio overestimates the risk ratio, we also estimated risk ratios: the fully adjusted disability–mortality risk ratio was 3.0 (modified Poisson regression with robust standard errors; 95% CI: 2.5–3.7) and 3.2 by marginal standardization, corresponding to an absolute increase of 8.2 percentage points in three-year mortality; the severe-disability risk ratio was 4.4 (Supplementary Table S10). The association therefore remains substantial on the risk-ratio scale, though smaller than the odds ratio implies.

Notably, social security type was not independently associated with mortality in this older cohort (non-contributory OR = 0.89, *p* = 0.32; unaffiliated OR = 1.11, *p* = 0.52), suggesting that nominal coverage was not associated with lower mortality in adjusted models. This null association was stable across less-selected subgroups (excluding severe baseline disability, OR = 0.90; excluding baseline chronic conditions, OR = 0.97; Supplementary Table S12), arguing against its being merely an artifact of survivor selection on baseline severity; however, the corresponding E-value of 1.31 indicates that the estimate is also compatible with a small true protective effect, so we interpret it cautiously (see Section 4.5). Female sex was strongly protective (OR = 0.48; 95% CI: 0.39–0.58; *p* < 0.001), while having any chronic condition at baseline independently doubled mortality risk (OR = 2.65; 95% CI: 2.12–3.35; *p* < 0.001). The best-fitting model (M5, severity gradient; AIC = 3,361) demonstrated that disability severity, rather than disability as a binary construct, captures the strongest mortality signal (full M4 coefficients in Supplementary Table S8).

### 3.6 Geographic overlap: disability, social protection, and healthcare infrastructure

Municipal-level analysis revealed that 1,728 of 2,469 municipalities (70.0%) had zero hospital beds (healthcare deserts), 403 (16.3%) had limited capacity ( ≤ 30 beds), and only 338 (13.7%) had adequate infrastructure (>30 beds). Among the 6.8 million PWD, 21.5% resided in healthcare deserts, 13.9% in limited municipalities, and 64.6% in adequate ones. The proportion of PWD in insufficient infrastructure (desert + limited: 35.4%) exceeded that of the general population (33.6%) by 1.9pp (*p* < 0.001; full census population). Even among municipalities classified as adequate, the median hospital bed density was 0.92 per 1,000 population.

**Figure 4:**
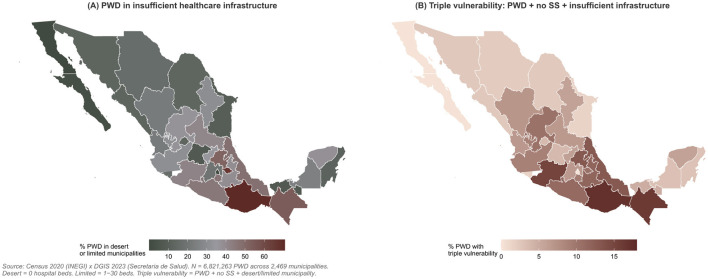
Geographic overlap of disability, healthcare infrastructure deficits, and social protection gaps. **(A)** Proportion of PWD residing in municipalities with insufficient healthcare infrastructure (desert or limited: ≤30 hospital beds) by state. Southern and central states show the highest concentration. **(B)** Triple vulnerability (PWD + no SS affiliation + insufficient infrastructure) by state. Oaxaca (17.2%), Chiapas (16.3%), and Michoacán (15.1%) have the highest proportions of triple-vulnerable PWD, corresponding to states with high marginalization indices. Source: Census 2020 (INEGI) × DGIS 2023 (Secretaría de Salud). *N* = 6,821,263 PWD across 2,469 municipalities. PWD, persons with disabilities; SS, social security.

Triple vulnerability, the intersection of disability, lack of SS affiliation, and residence in a municipality with insufficient healthcare infrastructure, was estimated to affect approximately 502,858 individuals (7.4% of all PWD; 37.2% of unaffiliated PWD). Because this figure combines individual-level Census attributes (disability and affiliation) with a municipality-level infrastructure classification, it is an ecological estimate of how many PWD reside in under-resourced municipalities rather than an individually verified count of persons lacking access to care. Geographic concentration was marked (Figure 4): Oaxaca (17.2% of state PWD), Chiapas (16.3%), and Michoacán (15.1%) had the highest proportions of triple-vulnerable PWD, corresponding to states with high marginalization indices and large indigenous populations. In absolute terms, Veracruz (63,701), Estado de México (54,979), and Oaxaca (49,152) had the largest concentrations. These findings identify specific geographic targets where social protection, healthcare infrastructure, and disability-inclusive interventions converge as policy priorities (Supplementary Figure S4).

## 4 Discussion

### 4.1 Summary of principal findings

This study provides one of the first multi-source, population-level analyses linking disability to social protection, chronic disease morbidity, and prospective mortality in Mexico. Drawing on nine nationally representative data sources encompassing over 125 million persons, five principal findings emerged: (1) confounding by age structure in social security coverage—an amalgamation effect of the kind formalized in Simpson's paradox—whereby PWD appeared better covered in aggregate (19.8% vs. 22.9% unaffiliated) but showed null association after age adjustment (OR = 0.99) together with a structural shift from contributory to non-contributory coverage; (2) substantial economic consequences including significantly higher catastrophic health expenditure (crude ratio 2.3; adjusted OR = 2.02) and widening retirement savings gaps; (3) significantly higher prevalence of all nine conditions assessed, with adjusted ORs surviving Benjamini–Hochberg correction and less than 5% attenuation after socioeconomic adjustment; (4) a fully adjusted prospective mortality OR of 3.77 with a dose–response severity gradient reaching 7.24 for severe disability; and (5) geographic concentration of 502,858 triple-vulnerable PWD in states with high marginalization and large indigenous populations.

Two apparent contradictions are central to this study's contribution. First, the *coverage paradox*: PWD appear better covered in aggregate statistics, but this reflects non-contributory enrollment driven by the older age structure of the PWD population, not genuine protective inclusion (57). Second, the *protection paradox*: even when PWD hold social security affiliation, non-contributory coverage was not associated with lower mortality (OR = 0.89, *p* = 0.32), consistent with the systematic review conclusion that health insurance effects on PWD in LMICs are “limited and inconclusive” (13, 14). Together, these paradoxes reveal a system characterized by *apparent inclusion but functional exclusion*.

### 4.2 Social security coverage and confounding by age structure

The confounding by age structure documented here [an amalgamation effect of the kind described by Simpson's paradox (57)] has direct policy implications. Because 46% of PWD are aged 60 or older (vs. 10% of the general population), and older adults have near-universal enrollment through non-contributory programs, aggregate coverage statistics fundamentally misrepresent the situation of working-age PWD. Within the 30–44 age group, PWD had marginally higher exclusion rates than their non-disabled counterparts, though the pattern varied across older groups as non-contributory programs differentially absorbed PWD. This aggregation bias is particularly consequential because policymakers relying on aggregate data would conclude that no coverage gap exists, when in fact the gap is masked by the older age structure of the PWD population. The progressive logistic models (M1 to M4) make this mechanism transparent; the unadjusted OR of 0.82 (apparent protection) attenuates to a null association (OR = 0.99) after demographic adjustment, indicating that age confounding is the primary explanation.

The institutional composition of coverage reinforces this interpretation. The structural shift from contributory (employment-based) to non-contributory programs reflects labor market exclusion: with an employment rate of 24.9% (vs. 42.3% for non-disabled), PWD are systematically excluded from IMSS and ISSSTE coverage that provides comprehensive benefits packages. The ILO has documented that PWD earn 12% less per hour globally and 26% less in LMICs, and are overrepresented in informal employment lacking social security contributions (15, 16). Our finding that the AFORE retirement savings gap widens progressively with age, from 4.9pp at ages 18–29 to 10.6pp at ages 45–59, signals a cumulative disadvantage trajectory that may contribute to pension poverty risk for working-age PWD, potentially reinforcing the disability–poverty association documented across LMICs (9, 61).

The substantially higher crude prevalence of catastrophic health expenditure (2.8% vs. 1.2%, ratio 2.3), supported by a fully adjusted OR of 2.02, aligns with the global evidence on disability-related extra costs, which include assistive devices, specialized care, home modifications, and personal assistance (17). The modest attenuation from unadjusted (OR ≈ 2.4) to fully adjusted (OR ≈ 2.0) indicates that the disability effect operates largely independently of income level and household composition, consistent with the “extra costs” framework rather than being a mere proxy for poverty (18). The sharpest disparity in the poorest quintile (11.6% vs. 5.7%) indicates that catastrophic spending is most prevalent where it is least affordable, compounding the multidimensional poverty burden documented by Pinilla-Roncancio for PWD across Latin America (62).

### 4.3 Intersectional exclusion

The finding that disability amplifies disadvantage for indigenous persons (interaction OR = 1.10) and women (interaction OR = 1.07) provides empirical quantification of intersectional theory in a large-scale LMIC population. This goes beyond documenting that multiple disadvantages accumulate: it suggests that the combination is associated with effects *exceeding* the sum of parts—the hallmark of intersectional rather than merely additive disadvantage (20, 21). We distinguish the two scales explicitly: on the absolute (percentage-point) risk scale the excess over the sum of individual penalties constitutes a super-additive effect, whereas on the multiplicative odds scale the corresponding M5 interaction terms (1.07 and 1.10) represent supermultiplicative departures. These are complementary descriptions of the same phenomenon on different scales. Our findings align with Bixby et al. (19), who reported similar non-additive patterns at the intersection of disability, gender, and race/ethnicity in the United States (19), and extend this framework to indigenous identity, a dimension particularly relevant for Latin America where approximately 8.5% of the population identifies as indigenous.

The age-stratified pattern (widening gaps during working age, from 4.0pp at 15–29 to 6.6pp at 45–59, and narrowing at 60+ due to non-contributory coverage) implies that the most critical intervention window is during prime working years. For indigenous women with disability, the most multiply marginalized profile, the super-additive penalty substantially attenuates the residual protective effect observed for other PWD subgroups. This has direct implications for Mexico's General Law for the Inclusion of Persons with Disabilities (LGIPD), which mandates protections for this population (39), and for CONEVAL's poverty measurement framework, which does not incorporate disability as a measurement dimension—although it reports poverty among PWD as a population group—despite the documented intersectional compounding (38).

### 4.4 Chronic disease excess and social deprivations

The 84.1% CES-D depression screen prevalence among PWD exceeds the upper bound of the 8%–66% range reported in the global scoping review by Asdaq et al. (25). This likely reflects three factors: (a) the CES-D 7-item screen captures depressive symptomatology rather than clinical diagnosis, with 83.3% sensitivity and 90.2% specificity validated in Mexican older adults (63) (vs. 22.6% for physician-diagnosed depression, more aligned with the literature); (b) the disability definition includes mental health conditions, inflating the association; and (c) limited access to mental health services in Mexico; globally, unmet mental health needs among PWD have been estimated at 4.5–7.2 times those of the general population (30). Notwithstanding these caveats, the fully adjusted OR of 2.92 for depressive symptomatology and 2.23 for diagnosed depression are consistent with the umbrella review finding that 86% of pooled estimates demonstrate significant disability–health associations (22).

Chronic kidney disease (OR = 2.57) and multimorbidity (OR = 2.25) align with global scoping reviews reporting elevated odds ratios for chronic diseases among PWD (23, 24). The less than 5% attenuation after education and indigenous language adjustment is noteworthy: it suggests disability is independently associated with chronic disease, not merely a proxy for socioeconomic disadvantage. This aligns with the “cascade of disparities” framework, whereby higher exposure to adverse conditions, inadequate preventive care, and delayed treatment are associated with worse outcomes (32). Critically, the corroboration of morbidity excess across two independent data sources (ENSANUT (51) for adults 20+ and ENASEM (52) for adults 50+) with different sampling frames and measurement instruments strengthens confidence in the associations, constituting a form of conceptual replication. In Mexico specifically, Rivera-Almaraz et al. documented elevated multimorbidity among older adults with functional limitations in the ENASEM cohort (26), and similar patterns have been reported in India using SAGE Wave-2 data (64), suggesting these associations are robust across LMIC contexts.

The finding that education shows the strongest gradient for social security exclusion, with higher education conferring the largest reduction in exclusion odds relative to no schooling (Supplementary Table S9), positions education as the most modifiable social determinant. This fills an important gap: Hessel et al. (43) documented educational inequalities in disability-linked social security coverage across five Latin American countries (Chile, Colombia, El Salvador, Paraguay, and Uruguay), but Mexico was notably absent from that analysis (43). Our study confirms that the educational gradient observed in those countries extends to Mexico, and that the gradient operates through the same mechanism: formal employment requiring educational credentials provides access to contributory social security, as previously described by Saenz et al. for disability onset pathways (41).

### 4.5 Prospective mortality and the protection paradox

Our fully adjusted OR of 3.77 for three-year mortality substantially exceeds the pooled hazard ratio of 2.02 (95% CI: 1.77–2.30) from the global meta-analysis of 70 cohort studies by Smythe and Kuper (27). Several factors may explain this discrepancy: (a) our measure captures ADL and IADL limitations (functional disability), which tends to select more severe cases than self-reported impairment measures used in many included studies; (b) the ENASEM (52) cohort is aged 50+, where disability–mortality associations are typically stronger than in younger populations; (c) the three-year follow-up may capture more proximal deaths than longer follow-ups that dilute the effect through competing risks; and (d) Mexico-specific contextual factors (including healthcare access barriers, poverty, and the functional exclusion documented here) may amplify the disability–mortality association compared to the mix of predominantly high-income countries in the meta-analysis. Our severity gradient (mild OR = 1.89, moderate 3.14, severe 7.24) closely parallels the HUNT study from Norway, where severe motor disability yielded a hazard ratio of 3.67 over 35 years of follow-up (28). That our severe-disability OR (7.24) exceeds this comparator from a high-income country with universal healthcare underscores the additional mortality burden associated with Mexico's fragmented health system. A prior ENASEM analysis by Rivera-Almaraz et al. reported disability–mortality associations in the 2001–2012 waves, but did not adjust for social security type or examine severity gradients (26); our study extends this work with the most recent waves and a more comprehensive adjustment strategy.

The finding that social security type was not independently associated with mortality in this older cohort (non-contributory OR = 0.89, *p* = 0.32; unaffiliated OR = 1.11, *p* = 0.52) warrants careful interpretation. This result is restricted to adults aged 50+ and may be influenced by survivor selection (individuals who died before the 2018 baseline are excluded), differences in baseline severity not fully captured by our covariates, and unmeasured confounders related to effective healthcare access and utilization. As reported above (Supplementary Table S12), this null association persisted when the most selection-prone subgroups were excluded, indicating it is not driven solely by differential early mortality among the most severely affected PWD, who would have been most likely to die before the 2018 baseline. The estimate is nonetheless fragile: residual left-truncation would bias it toward the null, and its modest associated E-value indicates that a relatively weak unmeasured factor could be compatible with a small protective effect that our design would not detect. We therefore cannot exclude a true protective association of non-contributory coverage and make no causal claim about it. Rather than a definitive conclusion about health system effectiveness, this finding should be understood as evidence questioning the sufficiency of nominal coverage to produce measurable mortality differences in this population—that is, enrollment alone does not appear to translate into differential survival in these data. This interpretation aligns with the systematic review conclusion that health insurance effects on PWD in LMICs are “limited and inconclusive” (13), and with van Gameren and Enciso, who reported mixed evidence regarding the effect of Mexico's Seguro Popular on disability progression among older adults with chronic degenerative diseases (42). A plausible mechanism is the “cascade of disparities”: nominal enrollment may not overcome physical accessibility barriers, attitudinal discrimination from providers, lack of disability-trained personnel, and geographic distance to facilities, barriers documented by 42% of PWD in the ENADIS 2022 (49) disability module. Building disability-inclusive health systems likely requires structural changes beyond enrollment expansion (31).

The clear dose–response gradient across disability severity levels, captured by the best-fitting severity model (Supplementary Tables S8, S14), demonstrates that disability is not a monolithic category. Binary disability measures, while useful for prevalence estimation, mask important heterogeneity in mortality risk. Future surveillance should adopt severity-graded classifications using instruments such as the Washington Group Extended Set (6), which would enable targeted interventions proportional to need.

### 4.6 Geographic disparities and triple vulnerability

The finding that 70% of Mexico's municipalities are healthcare deserts (zero hospital beds) identifies a structural barrier that social security enrollment alone cannot address. Even among municipalities classified as “adequate” (>30 beds), the median bed density of 0.92 per 1,000 falls below Mexico's national average of 1.0—itself the lowest among OECD member countries, less than one-quarter of the OECD average of 4.3 beds per 1,000 (65). For the 502,858 triple-vulnerable PWD, barriers are compounded: no coverage to pay for care, and limited local inpatient capacity even where coverage exists. The geographic concentration in Oaxaca (17.2%), Chiapas (16.3%), and Michoacán (15.1%), states with high marginalization indices and large indigenous populations, reinforces the intersectional findings and identifies a “disability disadvantage belt” across southern Mexico (33).

It is important to note that these geographic associations are ecological in nature, derived from merging individual-level census records with municipal-level infrastructure indicators; individual-level inferences about healthcare access cannot be drawn directly from these aggregated data. Rather than uniform national policies, the geographic analysis provides an actionable targeting framework primarily for descriptive and planning purposes—oriented toward territorial prioritization of interventions: resources should be concentrated in the approximately 500 municipalities with the highest triple vulnerability scores. This approach aligns with the CRPD's requirement for disaggregated disability data to guide policy (7), supports monitoring of the country's commitments under the 2030 Agenda for Sustainable Development (8), and responds to calls for building disability-inclusive health systems from the ground up (31). The convergence of disability, social security exclusion, and infrastructure deficits in the same municipalities suggests that single-axis interventions, such as expanding enrollment alone, will be insufficient; multi-sectoral approaches addressing infrastructure, coverage quality, and accessibility simultaneously are required.

### 4.7 Novelty and implications for Latin America

This study makes several contributions that are, to our knowledge, novel. First, no prior country-level study has documented this age-structure confounding in disability–social security coverage, providing a methodological cautionary note for aggregate monitoring indicators. Second, it represents one of the first multi-source analyses (nine databases, >125 million persons) integrating social protection, morbidity, and mortality outcomes for PWD in the Latin American context. Third, it fills the conspicuous Mexico gap in Hessel et al.'s five-country LAC analysis of educational inequalities in disability-linked social security (43). Fourth, it provides one of the first quantitative intersectional analyses of disability × indigenous identity × gender on social security exclusion in Mexico. Fifth, it generates one of the first prospective mortality estimates with a severity gradient for PWD in Mexico using nationally representative data.

These findings have broad implications for the estimated 85 million PWD across Latin America and the Caribbean (33, 34), a region where 76% of quantitative studies on healthcare access for PWD have medium or high risk of bias (33). The multidimensional poverty of PWD in LAC has been documented (62), but the mechanisms linking social protection gaps to health outcomes remain poorly elucidated. Our study demonstrates that open government data approaches can produce rigorous, multi-source evidence without primary data collection (66), a replicable model for other countries in the region.

### 4.8 Limitations

Several limitations should be considered when interpreting these findings. First, most analyses are cross-sectional, precluding causal inference; the prospective ENASEM component partially addresses this but covers only adults aged 50+. Second, no individual-level record linkage was attempted across independent cross-sectional surveys, as all sources are anonymized with non-overlapping sampling frames; each survey was analyzed independently, and cross-source comparisons are ecological in nature (the ENASEM prospective component uses within-survey panel linkage). Third, all disability measures rely on self-report, which may underestimate disability prevalence among individuals with cognitive limitations or cultural reluctance to report functional difficulties; however, the Washington Group methodology employed across sources is the international gold standard for comparable disability statistics (6). Fourth, although the integration of multiple data sources spanning 2017 [ENESS (50)] to 2023 [DGIS (54)] constitutes a strength by offering a comprehensive, complementary view of the phenomenon, this period encompasses significant structural changes in Mexico's health system, including the dissolution of Seguro Popular, the creation and subsequent dissolution of INSABI, and the ongoing transition to IMSS-Bienestar (44). Our results therefore reflect a system in transition, and conclusions should be interpreted within the context of the specific measurement period of each source. This window also encompasses the COVID-19 pandemic (2020–2021), which may have differentially affected PWD in terms of social exclusion, healthcare access, and mortality; with the exception of the ENASEM panel, each source is a single cross-section, so we treat the 2017–2023 span as a set of complementary contemporaneous snapshots rather than a directly comparable time series, and pandemic-related period effects cannot be separated from secular trends. Future evaluations will be necessary to determine whether the patterns documented here—particularly the protection paradox and the dominance of non-contributory coverage—persist, improve, or worsen under the new care model. Fifth, mortality models use logistic regression yielding odds ratios rather than survival analysis yielding hazard ratios; given the 3-year mortality rate of 18.1% among PWD, some overestimation of the risk ratio is possible, though the direction and significance of associations would be preserved. Risk-ratio estimates reported above (Supplementary Table S10) confirm this: the disability–mortality association remained large and highly significant on the risk-ratio scale, though smaller than the odds ratio implies. We therefore report odds ratios in the main text for consistency with the other analyses, while cautioning that they overstate the relative risk at this event rate.

Sixth, the 84.1% CES-D depression screen prevalence should be interpreted as depressive symptomatology rather than clinical depression; the 22.6% physician-diagnosed rate provides a more conservative estimate. Seventh, the healthcare infrastructure analysis merges ecological municipal-level data with individual-level records, and within-municipality variation in healthcare access is not captured; consequently, individual-level inferences should not be drawn from these ecological associations, and the geographic findings are intended primarily for descriptive purposes and territorial prioritization of interventions rather than for individual-level causal inference. Eighth, survivor bias in the ENASEM (52) cohort may attenuate mortality estimates, as individuals who died before the 2018 baseline are excluded; our ORs may therefore be conservative. Ninth, and critically, the disability definitions across sources encompass heterogeneous constructs: the Census (45) and ENSANUT (51) employ Washington Group-based functionality scales, ENASEM (52) relies on ADL/IADL limitations capturing a different dimension of disability, ENIGH (48) uses an inverted scale requiring careful recoding, and CONEVAL (46) provides a pre-calculated binary indicator. These instruments do not capture exactly the same construct: functionality-based measures (WG) emphasize activity limitations across domains, while ADL/IADL scales focus on dependence in specific daily tasks, and administrative variables may reflect institutional rather than clinical criteria. This heterogeneity may influence the magnitude of observed associations and limits direct comparability across analyses. Accordingly, our findings should be interpreted as a convergence of evidence under different operationalizations of disability—each internally consistent—rather than as a homogeneous measurement of a single phenomenon. The consistency of the direction and significance of associations across these diverse instruments strengthens the overall conclusions, but the precise effect sizes are not directly comparable between sources. A sensitivity analysis varying the Census disability threshold confirmed that the main logistic model results were robust across a wide prevalence range (Supplementary Table S3).

Despite these limitations, several design features strengthen confidence in our findings: triangulation across nine independent data sources with broadly consistent patterns, progressive model specification making confounding adjustment transparent, Benjamini–Hochberg correction for multiple comparisons, and convergent morbidity results across ENSANUT (51) and ENASEM (52), datasets with different sampling frames, measurement instruments, and target populations. This cross-source convergence constitutes a form of conceptual replication that is rarely achieved in single-source studies.

### 4.9 Strengths and future directions

Key strengths of this study include its population scale (125.5 million expanded population), integration of nine nationally representative sources covering complementary dimensions of the disability experience, multi-dimensional assessment spanning social protection, morbidity, mortality, and geographic access, methodological transparency through nested models, intersectional design extending beyond single-axis analyses, exclusive use of open government data, and a prospective cohort component reducing reverse causation concerns for the mortality analysis.

Several future research priorities emerge from these findings. First, linked data infrastructure enabling longitudinal tracking of PWD across social security enrollment, healthcare utilization, and health outcomes would allow causal analyses currently precluded by the cross-sectional design. Second, our findings provide a pre-reform baseline against which the ongoing IMSS-Bienestar transition can be evaluated; determining whether the restructuring improves or worsens effective coverage for PWD is a critical policy question. Third, disaggregation by disability type (sensory, motor, cognitive, psychosocial) would reveal heterogeneity masked by the binary disability measure. Fourth, qualitative research is needed to understand the mechanisms behind the “nominal coverage without mortality protection” finding, specifically what happens between enrollment and health outcome that breaks the expected protective pathway. Fifth, cost-effectiveness analyses of disability-inclusive social security reforms would inform resource allocation. Finally, replication of this multi-source open data approach across other Latin American countries (31, 40) would establish whether the patterns documented here, particularly the age-structure confounding and the protection paradox, are specific to Mexico or reflect broader regional dynamics.

## 5 Conclusions

This study demonstrates that disability in Mexico carries a compounding burden across social protection, health, and survival. The apparent social security advantage of PWD is largely an artifact of their older age structure: a shift toward non-contributory programs confers nominal affiliation without effective protection, and the modest residual advantage is erased—and reversed—for the most multiply marginalized groups, notably indigenous women with disability. Across independent sources, PWD also faced higher catastrophic health expenditure, a greater burden of chronic disease and depressive symptoms, and elevated, severity-graded mortality. Non-contributory affiliation was not associated with lower mortality; this observational estimate cannot support causal inference but is consistent with a gap between nominal enrollment and effective protection that warrants prospective investigation.

Three implications follow: aggregate coverage statistics misrepresent PWD and call for age-stratified, institution-disaggregated monitoring; expanding enrollment is likely insufficient on its own, making the ongoing transition to IMSS-Bienestar an opportunity to embed disability-inclusive design rather than re-enroll the same population under a new banner; and the triple-vulnerability framework provides a concrete tool for geographically targeting the municipalities where disability, coverage gaps, and infrastructure deficits converge. Together, these findings offer an empirical baseline for monitoring Mexico's commitments to persons with disabilities under the Convention on the Rights of Persons with Disabilities and the 2030 Agenda for Sustainable Development.

## Data Availability

The datasets presented in this study can be found in online repositories. The names of the repository/repositories and accession number(s) can be found in the article/Supplementary material.
