# Supplementary material for: The compounding burden of disability in Mexico: social protection gaps, chronic disease excess, and prospective mortality from national open data sources
Source: Front Public Health. 2026 Jul 2;14:1847293. doi: 10.3389/fpubh.2026.1847293 (PMC13373609; doi:10.3389/fpubh.2026.1847293)
Supplement: Supplementary file 1 [file Data_Sheet_1.PDF]

Supplementary Material: The compounding burden of disability in Mexico

Contents

Table S1. Data sources, sample sizes, and disability operationalization 2
Table S2. Key variables used in analyses, by data source 3
Table S3. Sensitivity analysis of the full logistic model (M4) under alternative disability thresholds, Census 2020. 7
Table S4. Social security indicators by disability status, ENESS 2017 (N = 259,673). 8
Table S5. Nested logistic regression models for catastrophic health expenditure by household disability status, ENIGH 2022 (N = 90,102 households). 9
Table S6. Fully adjusted odds ratios for health outcomes by disability status, ENASEM 2021 (N = 15,739 adults aged 50+). 10
Table S7. Three-year mortality by baseline disability severity, ENASEM 2018–2021. 11
Table S8. Full coefficients of the prospective mortality models M1–M4, ENASEM 2018–2021 (model-specific Ns: 10,268 for M1–M2, 10,169 for M3–M4). 12
Table S9. Full coefficients of nested logistic regression models (M1–M5) for lack of social security affiliation, Census 2020. 13
Table S10. Risk ratios for prospective three-year mortality by disability status, ENASEM 2018–2021. 14
Table S11. ENIGH 2022 disability prevalence and sensitivity of the catastrophic-expenditure association to the disability threshold. 15
Table S12. Survivor- and selection-bias sensitivity analyses for the prospective mortality models. 16
Table S13. Fit statistics of the nested survey-weighted logistic models for lack of social security affiliation (M1–M5), Census 2020. 17
Table S14. Nested logistic regression models for prospective three-year mortality, ENASEM 2018–2021. 18
Figure S1. Disease prevalence by disability status 19
Figure S2. Adjusted odds ratios for the assessed conditions: PWD vs. no disability 20
Figure S3. Inter-wave mortality rate by age group and sex, ENASEM 2018–2021 21
Figure S4. Triple vulnerability among PWD by state (dot plot) 22

Table S1. Data sources, sample sizes, and disability operationalization

Source	Year	Institution	N (sample)	N (PWD)	Disability criterion	Survey design
Census 2020 (Extended)	2020	INEGI	15,015,683	∼6.8M	DIS_* $\geq$ 3 or DIS_MENTAL = 5	ESTRATO / UPM / FACTOR
CONEVAL 2022	2022	CONEVAL	309,534	21,846	discap = 1 (pre-calculated)	est_dis / upm / factor
ENIGH 2022	2022	INEGI	309,684	∼21,848	disc_* $\leq$ 2 (inverted scale)	est_dis / upm / factor
ENADIS 2022	2022	INEGI	5,696	5,696	Pre-selected PWD module	EST_DIS / UPM_DIS / FAC_DIS
ENESS 2017	2017	INEGI	259,949	Variable	AFI_DISCA = 1 or 2	D_SEM / UPM / FACTOR
ENSANUT 2022	2022	INSP	11,913	1,309	WG Short Set $\geq$ 3 (aided)	estrato / upm / ponde_f
ENASEM 2018–2021	2018/ 2021	INEGI/ UTMB	15,739 (living 2021)	3,252	N_ABVD $\geq$ 1 or N_AIVD $\geq$ 1	EST_DIS / UPM_DIS / FACTORI
CONAPO 2020	2020	CONAPO	2,469 munic.	—	N/A	N/A (administrative)
DGIS 2023	2023	SS	21,624 facilities	—	N/A	N/A (administrative)

INEGI = Instituto Nacional de Estadística y Geografía; CONEVAL = Consejo Nacional de Evaluación de la Política de Desarrollo Social; ENIGH = Encuesta Nacional de Ingresos y Gastos de los Hogares; ENADIS = Encuesta Nacional sobre Discriminación; ENESS = Encuesta Nacional de Empleo y Seguridad Social; ENSANUT = Encuesta Nacional de Salud y Nutrición; INSP = Instituto Nacional de Salud Pública; ENASEM = Estudio Nacional de Salud y Envejecimiento en México; MHAS = Mexican Health and Aging Study; UTMB = University of Texas Medical Branch; CONAPO = Consejo Nacional de Población; DGIS = Dirección General de Información en Salud; SS = Secretaría de Salud; WG = Washington Group; ADL = Activities of Daily Living; IADL = Instrumental ADL.

Table S2. Key variables used in analyses, by data source

A. Census 2020 — Disability and social security

Variable	Type	Description	Values / Scale
DIS_VER	int	Difficulty seeing, even with glasses	1=None, 2=Some, 3=A lot, 4=Cannot
DIS_OIR	int	Difficulty hearing, even with hearing aid	Same scale
DIS_CAMINAR	int	Difficulty walking or climbing stairs	Same scale
DIS_RECORDAR	int	Difficulty remembering or concentrating	Same scale
DIS_BANARSE	int	Difficulty bathing, dressing, or eating	Same scale
DIS_HABLAR	int	Difficulty communicating	Same scale
DIS_MENTAL	int	Mental health condition	5=Has condition, 6=No
DHSERSAL1	char	Primary health service affiliation	01=IMSS, 02=ISSSTE, 05=INSABI, 06=IMSS-Bien., 07=Private, 09=Unaff.
SEXO	char	Sex	1=Male, 3=Female
EDAD	int	Age in years	Continuous
ESCOACUM	char	Accumulated years of schooling	0–24, 99=NS (no leading zeros)
PERTE_INDIGENA	char	Indigenous self-identification	1=Yes, 2=Partially, 3=No
HLENGUA	char	Indigenous language speaker	1=Yes, 3=No
TAMLOC	char	Locality size	1=$<$2,500 to 4=$\geq$100,000
ENT, MUN	char	State and municipality codes	2-digit, 3-digit (zero-padded)
ESTRATO, UPM, FACTOR	—	Survey design variables	Stratum, PSU, weight

B. CONEVAL 2022 — Social deprivations and poverty

Variable	Type	Description	Values
discap	int	Disability status (pre-calculated)	0=No, 1=Yes
ic_segsoc	int	Social security deprivation	0=No, 1=Deprived
ic_asalud	int	Health services deprivation	0/1
ic_rezedu	int	Educational lag	0/1
ic_cv	int	Housing quality deprivation	0/1
ic_sbv	int	Basic housing services deprivation	0/1
ic_ali	int	Food security deprivation	0/1
pobreza	int	In poverty (any type)	0/1
carencias	int	Count of social deprivations	0–6

C. ENSANUT 2022 — Chronic conditions and depression screening

Variable	Type	Description	Values
a1403a–a1408	int	WG Short Set domains (6)	1–4 (aided for vision/hearing)
a0301	int	Diabetes diagnosis	1=Yes
a0401	int	Hypertension diagnosis	1=Yes
a0202	int	Depression diagnosis	1=Yes
a0502a–d	int	CVD events (MI, angina, HF, stroke)	1=Yes each
a0601c	int	Chronic kidney disease	1=Yes
a0604	int	High cholesterol	1=Yes

a0211–a0217	int	CES-D 7-item depression scale	1–4 (a0216 reversed)
H0310A	char	Primary health affiliation	01–11
h0317a	int	Education level	0–12
h0311	int	Indigenous language speaker	1=Yes, 2=No

D. ENASEM 2018–2021 — ADL/IADL, mortality, and health outcomes

Variable	Type	Description	Values
N_ABVD_18/21	int	Basic ADL limitation count	0–5
N_AIVD_18/21	int	Instrumental ADL limitation count	0–4
EDUCACION	int	Years of education	0–22
FALLECIDO_21	int	Died between 2018–2021	1/2=Died, 0=Alive
SEGURO_MEDICO	int	Any health insurance	1=Yes
IMSS/ISSSTE/INSABI	int	Specific affiliations (2021)	1=Yes each
SEG_POP_18	int	Seguro Popular (2018 baseline)	1=Yes
N_ENF_18/21	int	Chronic disease count	0–7
HOSPITALIZACION_21	int	Hospitalized past 12 months	1=Yes
GENERO	int	Sex	1=Male, 2=Female

E. ENIGH 2022 — Health expenditure and employment benefits

Variable	Type	Description	Values / Scale
disc_camin	int	Walking difficulty (inverted scale)	1=Cannot, 2=Great diff., 3=Some, 4=None
disc_ver, disc_oir, disc_brazo, disc_apren, disc_vest, disc_habla, disc_acti	int	7 additional disability domains	Same inverted scale
salud	num	Total quarterly health expenditure (MXN)	Continuous
ing_cor	num	Current quarterly household income (MXN)	Continuous
alimentos	num	Quarterly food expenditure (MXN)	For capacity-to-pay calculation
pres_8	char	SAR/AFORE retirement savings	Non-empty = has AFORE
contrato	int	Employment contract type	1=Written, other=Informal
folioviv, foliohog, numren	char	Household and person identifiers	Join keys across ENIGH tables
est_dis, upm, factor	—	Survey design variables	Stratum, PSU, weight

F. ENADIS 2022 — Access barriers and discrimination

Variable	Type	Description	Values
PM2_7_1–7	int	Insufficient preparation at 7 institutions	1=Sufficient, 2=Insufficient
PM9_1_1–8	int	Denial of rights in past 5 years (8 domains)	1=Denied, 2=Not denied
PM2_2	int	Main perceived problem faced by PWD	1–9 categorical
PM9_6_12	int	Discrimination due to disability (12 months)	1=Yes, 2=No
SEXO, EDAD	int	Sex and age	1=Male; continuous
EST_DIS, UPM_DIS, FAC_DIS	—	Survey design variables	Stratum, PSU, weight

G. ENESS 2017 — Employment and social security

Variable	Type	Description	Values
AFI_DISCA	int	Disability/limitation status	0=None, 1=Limitation, 2=Disability
AFI_CON	int	SS affiliation status	1=Affiliated, 2=Not
AFI_MED	int	Affiliation institution	1=IMSS, 2=ISSSTE, 3=Seguro Popular, 6=Unaffiliated
PENSION	int	Receives pension	1=Yes, 2=No
PEN_TIP	int	Pension type	1=Retirement, 2=Disability, 3=Other
COT_ACT	int	Active contribution status	1=Contributing, 2=Not
P7_1–P7_8	int	Reasons for non-affiliation	Multiple response (PWD only)
P1_EDA, P1_SEX	int	Age and sex	Continuous; 1=Male
D_SEM, UPM, FACTOR	—	Survey design variables	Stratum, PSU, weight

H. CONAPO 2020 — Marginalization indices

Variable	Type	Description	Values
CVE_ENT	int	State code	1–32
CVE_MUN	int	Municipality code	Numeric (3-digit extracted)
IM_2020	num	Marginalization index 2020	Continuous
GM_2020	char	Marginalization grade 2020	Very high / High / Medium / Low / Very low
IMN_2020	num	Normalized marginalization index	0–100
POBLACION	num	Projected mid-year population 2020	Continuous

I. DGIS 2023 — Healthcare infrastructure

Variable	Type	Description	Values
Clave.Estado	int	State code	1–32
Clave.Municipio	int	Municipality code	Numeric
TOTAL.CAMAS.AREA.HOSP	int	Hospital beds	Count per facility
TOTAL.CAMAS.OTRAS.AREAS	int	Beds in other areas	Count per facility
desert_category	char	Healthcare desert classification	Desert (0) / Limited (1–30) / Adequate (>30)

J. Derived variables used in analyses

Variable	Source	Definition
tiene_discapacidad	Census	max(DIS_VER,...,DIS_HABLAR) $\geq$ 3 OR DIS_MENTAL = 5
sin_afiliacion	Census	DHSERSAL1 = "09" (no affiliation)
ss_type (4-cat)	Census	Contributory (01–03), Non-contributory (05–06), Private/Other (07–08), Unaffiliated (09)
escolaridad (5-cat)	Census	ESCOACUM as integer: 0, 1–6, 7–9, 10–12, 13–24
pwd_18	ENASEM	N_ABVD_18 $\geq$ 1 OR N_AIVD_18 $\geq$ 1

disability_sev_18	ENASEM	4 levels: No disability / Mild (IADL only) / Moderate (1–2 ADL) / Severe (3+ ADL)
wg_max	ENSANUT	pmax across 6 WG domains (aided for vision/hearing)
cesd_score	ENSANUT	Sum of 7 CES-D items (a0216 reversed); positive ≥ 9
multimorbidity	Multiple	Chronic condition count ≥ 2
gc_oms	ENIGH	OOP health spending $>$ 40% of capacity to pay
triple_vuln	Census $\times$ DGIS	Disability AND no SS AND desert/limited infrastructure

A complete variable dictionary for all nine data sources, including variables not used in this study, is available as a supplementary Excel file (variable_dictionary_complete.xlsx) and deposited in Zenodo (https://doi.org/10.5281/zenodo.19364771).

Table S3. Sensitivity analysis of the full logistic model (M4) under alternative disability thresholds, Census 2020.

The full model (M4: disability + sex + age group + education + indigenous identity + rural residence) was re-estimated under three disability definitions to assess robustness of the main finding. The "Standard" row replicates the primary analysis reported in Table 2 of the main text.

Disability threshold	N PWD	Prevalence (%)	OR	95% CI	p-value
Broad ($\geq$ 2: some difficulty or worse)	2,275,472	21.2	0.92	0.91–0.93	< 0.001
Standard ($\geq$ 3: a lot of difficulty or worse)[a]	775,614	7.2	0.92	0.90–0.93	< 0.001
Strict ($=$ 4: cannot do it only)	171,353	1.6	0.86	0.84–0.88	< 0.001

OR: odds ratio; CI: confidence interval; PWD: persons with disabilities; SS: social security; WG: Washington Group. OR for lacking SS affiliation associated with disability, from survey-weighted logistic regression (quasibinomial, M4 specification). All thresholds include persons with mental health conditions (DIS_MENTAL = 5). N PWD and prevalence calculated on 10,739,396 eligible adults aged $\geq$15; effective model N is 10,717,409 after listwise exclusion of 21,987 records (0.2%) with missing education data, consistent with Table 2 of the main text.

[a]Primary analysis threshold (Washington Group criterion). Results approximate Table 2, M4 (minor variation due to automatic listwise deletion across thresholds).

The direction, magnitude, and statistical significance of the disability–social security association are consistent across a 13-fold range of disability prevalence (1.6% to 21.2%), with ORs ranging from 0.86 to 0.92. The stricter threshold yields a slightly stronger protective association (OR = 0.86), consistent with the dose–response pattern whereby more severe disability is associated with greater enrollment in non-contributory programs targeting older and more vulnerable populations.

Table S4. Social security indicators by disability status, ENESS 2017 (N = 259,673).

Indicator	Without disability (n = 239,053)	PWD (n = 20,620)	*p*
Key social security indicators, n (weighted %)			
SS affiliation	198,957 (82.4) [81.9–82.9]	18,269 (87.6) [86.8–88.4]	<0.001
Actively contributing to SS	45,983 (23.3) [22.7–23.8]	2,018 (9.1) [8.4–9.8]	<0.001
Receives any pension	8,291 (4.3) [4.1–4.5]	3,694 (17.6) [16.6–18.5]	<0.001
Reasons for non-affiliation (unaffiliated PWD only; n = 2,351)[a]			
No formal employment	—	1,001 (44.7) [41.4–48.0]	—
Unaware of requirements	—	1,037 (44.2) [40.9–47.6]	—
No nearby medical facility	—	372 (17.1) [14.6–19.7]	—
Costly procedure	—	320 (14.1) [11.8–16.4]	—
Poor quality of services	—	264 (11.2) [9.2–13.1]	—

Survey-weighted estimates (Rao–Scott χ^2 test). 95% CI in brackets. PWD = limitation (AFI_DISCA = 1) or disability (AFI_DISCA = 2). Active contribution: among PWD subcategories, 5.7% for those with limitation and 12.5% for those with disability. [a]Multiple response: percentages may sum to more than 100%. Reasons for non-affiliation were only collected from unaffiliated PWD in the ENESS questionnaire. Analytic N = 259,673 after exclusion of 276 records with missing affiliation or disability status (259,949 total). PWD: persons with disabilities; SS: social security; CI: confidence interval.

Table S5. Nested logistic regression models for catastrophic health expenditure by household disability status, ENIGH 2022 (N = 90,102 households).

A. WHO definition (>40% capacity to pay)

	M1			M2			M3		
**Term**	**OR**	**95% CI**	***p***	**OR**	**95% CI**	***p***	**OR**	**95% CI**	***p***
PWD household	2.39	2.07–2.76	<.001	2.13	1.84–2.46	<.001	2.02	1.73–2.35	<.001
Income Q2 (ref: Q1)	—	—	—	0.42	0.35–0.51	<.001	0.42	0.35–0.51	<.001
Income Q3	—	—	—	0.33	0.26–0.41	<.001	0.32	0.26–0.41	<.001
Income Q4	—	—	—	0.28	0.22–0.36	<.001	0.28	0.22–0.36	<.001
Income Q5	—	—	—	0.22	0.17–0.29	<.001	0.22	0.17–0.29	<.001
HH size	—	—	—	1.04	1.00–1.08	.039	1.04	1.00–1.08	.039
Elderly member	—	—	—	—	—	—	1.14	0.98–1.34	.096
Rural	—	—	—	—	—	—	1.67	1.44–1.95	<.001

B. Alternative definition (>10% income)

	M1			M2			M3		
**Term**	**OR**	**95% CI**	***p***	**OR**	**95% CI**	***p***	**OR**	**95% CI**	***p***
PWD household	2.41	2.20–2.64	<.001	2.35	2.14–2.57	<.001	1.96	1.78–2.16	<.001
Income Q2 (ref: Q1)	—	—	—	0.64	0.57–0.73	<.001	0.64	0.57–0.73	<.001
Income Q3	—	—	—	0.62	0.54–0.71	<.001	0.62	0.54–0.71	<.001
Income Q4	—	—	—	0.63	0.55–0.74	<.001	0.63	0.55–0.74	<.001
Income Q5	—	—	—	0.78	0.67–0.91	.002	0.78	0.67–0.91	.002
HH size	—	—	—	0.95	0.92–0.98	<.001	0.95	0.92–0.98	<.001
Elderly member	—	—	—	—	—	—	1.62	1.47–1.78	<.001
Rural	—	—	—	—	—	—	1.60	1.46–1.76	<.001

Survey-weighted logistic regression (quasibinomial). PWD household = at least one member with disability (ENIGH inverted scale ≤ 2). M1: unadjusted. M2: + income quintile, household size. M3: + elderly member, rural residence. OR attenuation from M1 to M3 is modest ($\sim$15%), suggesting disability is independently associated with catastrophic expenditure. OR: odds ratio; CI: confidence interval; PWD: persons with disabilities; HH: household.

Table S6. Fully adjusted odds ratios for health outcomes by disability status, ENASEM 2021 (N = 15,739 adults aged 50+).

Health outcome	OR_{adj}	95% CI	p_{adj}	N (PWD)
Stroke	4.12	2.78–6.11	<0.001	3,252
Depression (CES-D)	2.72	2.26–3.28	<0.001	3,252
Multimorbidity (≥2)	2.44	2.02–2.95	<0.001	3,252
Hospitalized (12 months)	2.31	1.81–2.95	<0.001	3,252
Heart problems	2.04	1.50–2.77	<0.001	3,252
Hypertension	1.90	1.59–2.26	<0.001	3,252
Diabetes	1.76	1.45–2.14	<0.001	3,252

Fully adjusted logistic regression: disability + age + sex + education + SS type. PWD = any ADL (N_ABVD $\geq$ 1) or IADL (N_AIVD $\geq$ 1) limitation. p_{adj} = Benjamini–Hochberg corrected. All outcomes significant after BH correction. Age-sex adjusted ORs showed <5% attenuation relative to fully adjusted models. Source: ENASEM/MHAS 2021 (INEGI/UTMB). OR: odds ratio; CI: confidence interval; PWD: persons with disabilities; SS: social security; ADL: activities of daily living; IADL: instrumental ADL; BH: Benjamini–Hochberg.

Table S7. Three-year mortality by baseline disability severity, ENASEM 2018→2021.

Disability severity (2018)	N	Deaths	Mortality (%)	*p*
No disability	9,308	324	3.5	ref
Mild (IADL only)	164	18	11.0	<0.001
Moderate (1–2 ADL)	571	81	14.2	<0.001
Severe (3+ ADL)	225	75	33.3	<0.001
**Total**	**10,268**	**498**	**4.9**	

p-values: χ^2 test vs. no disability (reference). Trend test $p < 0.001$. Source: ENASEM/MHAS 2018–2021 (INEGI/UTMB). N = 10,268 linked individuals; 498 deaths during 3-year follow-up. ADL: activities of daily living; IADL: instrumental ADL; PWD: persons with disabilities.

Table S8. Full coefficients of the prospective mortality models M1–M4, ENASEM 2018→2021 (model-specific Ns: 10,268 for M1–M2, 10,169 for M3–M4).

	M1		M2		M3		M4	
**Term**	**OR**	**95% CI**	**OR**	**95% CI**	**OR**	**95% CI**	**OR**	**95% CI**
Disability (PWD)	6.14	5.03–7.47	4.60	3.70–5.70	4.44	3.56–5.51	3.77	3.02–4.70
Age (per year)	—	—	1.06	1.05–1.08	1.06	1.05–1.08	1.06	1.05–1.08
Female	—	—	0.48	0.39–0.58	0.48	0.39–0.58	0.48	0.39–0.58
Educ (ref: None)								
Primary	—	—	—	—	0.79	0.59–1.06	0.79	0.59–1.06
Secondary	—	—	—	—	0.82	0.58–1.16	0.81	0.57–1.15
Upper sec+	—	—	—	—	0.55	0.38–0.80	0.54	0.37–0.79
SS type (ref: Contrib.)								
Non-contrib.	—	—	—	—	—	—	0.89	0.71–1.12
Other	—	—	—	—	—	—	1.31	0.75–2.17
Unaffiliated	—	—	—	—	—	—	1.11	0.80–1.51
Chronic condition	—	—	—	—	—	—	2.65	2.12–3.35
N	10,268		10,268		10,169		10,169	
AIC	3,725		3,473		3,456		3,384	

M = nested multivariate logistic regression model (unweighted, binomial). Disability: N_ABVD_18 ≥ 1 or N_AIVD_18 ≥ 1 at 2018 baseline. All disability ORs $p < 0.001$. Education: primary and secondary NS in M3–M4; upper secondary+ $p = 0.002$. SS type: all NS ($p > 0.3$). Chronic condition $p < 0.001$. AIC decreases from M1 to M4, confirming improved fit. M5 (severity gradient, AIC = 3,361) shown in Supplementary Table S14 and Figure 3. OR: odds ratio; CI: confidence interval; SS: social security; AIC: Akaike Information Criterion; ADL: activities of daily living; IADL: instrumental ADL; PWD: persons with disabilities.

Table S9. Full coefficients of nested logistic regression models (M1–M5) for lack of social security affiliation, Census 2020.

	M2		M3		M4		M5	
**Term**	**OR**	**95% CI**	**OR**	**95% CI**	**OR**	**95% CI**	**OR**	**95% CI**
Disability	0.99	0.98–1.01	0.92	0.91–0.93	0.92	0.90–0.93	0.87	0.85–0.88
Female sex	0.82	0.82–0.83	0.82	0.82–0.83	0.82	0.82–0.82	0.82	0.81–0.82
Age (ref: 15–29)								
30–44	0.83	0.82–0.84	0.83	0.82–0.84	0.83	0.82–0.83	0.83	0.82–0.83
45–59	0.71	0.71–0.72	0.72	0.71–0.73	0.72	0.71–0.73	0.72	0.71–0.73
60–74	0.47	0.47–0.48	0.47	0.46–0.48	0.47	0.46–0.48	0.47	0.46–0.47
≥75	0.42	0.41–0.42	0.41	0.40–0.42	0.41	0.40–0.42	0.41	0.40–0.42
Educ (ref: None)								
Primary	—	—	0.78	0.76–0.79	0.78	0.76–0.79	0.78	0.76–0.79
Lower sec	—	—	0.60	0.59–0.61	0.60	0.59–0.61	0.60	0.59–0.61
Upper sec	—	—	0.52	0.51–0.54	0.52	0.51–0.53	0.52	0.51–0.53
Higher ed	—	—	0.42	0.41–0.43	0.42	0.41–0.43	0.42	0.41–0.43
Indigenous	—	—	—	—	0.97	0.95–0.99	0.96	0.94–0.98
Rural	—	—	—	—	0.83	0.81–0.85	0.83	0.81–0.85
Disc × Female	—	—	—	—	—	—	1.07	1.04–1.09
Disc × Indig.	—	—	—	—	—	—	1.10	1.07–1.13
N	10,739,396		10,717,409		10,717,409		10,717,409	
Deviance	11,466,449		11,359,142		11,348,643		11,348,362	

Survey-weighted logistic regression (quasibinomial). All $p < 0.001$ except M2 disability ($p = 0.298$). M1 omitted (unadjusted, single term; OR = 0.82, see Table 2). Education shows the steepest gradient: higher education reduces SS exclusion odds by 58% relative to no schooling. M5 interactions indicate supermultiplicative associations (departures from multiplicativity on the odds scale) of disability × gender and disability × indigenous identity. OR: odds ratio; CI: confidence interval; SS: social security; PWD: persons with disabilities.

Table S10. Risk ratios for prospective three-year mortality by disability status, ENASEM 2018→2021.

Contrast	OR (95% CI)	$RR_{Poisson}$ (95% CI)	$RR_{g\text{-}comp}$ (95% CI)	Risk diff.
Disability (any ADL/IADL) vs. none	3.77 (3.02–4.70)	3.03 (2.49–3.68)	3.17 (2.61–3.85)	+8.2 pp
Severity gradient (vs. no disability):				
Mild (IADL only)	1.89 (1.07–3.15)	1.81 (1.14–2.85)	—	—
Moderate (1–2 ADL)	3.14 (2.37–4.14)	2.73 (2.16–3.44)	—	—
Severe (3+ ADL)	7.24 (5.18–10.0)	4.40 (3.39–5.70)	—	—

All models adjust for age, sex, education, social security type, and baseline chronic conditions (the M4/M5 specification; N = 10,169 with non-missing education; 498 deaths). OR: odds ratio from logistic regression (as reported in the main text). $RR_{Poisson}$: risk ratio from modified Poisson regression with robust (HC0 sandwich) standard errors. $RR_{g\text{-}comp}$: risk ratio from marginal standardization (g-computation) over the covariate distribution of the analytic sample, with a nonparametric bootstrap 95% CI (2,000 replicates); the corresponding marginal risk difference is shown. Because the three-year mortality among PWD is high (18.1% in the full linked cohort; 18.3% in this analytic sample), the odds ratio overestimates the risk ratio; the risk ratio of ∼3.0–3.2 (and 4.4 for severe disability) is the more interpretable measure of relative risk. OR: odds ratio; RR: risk ratio; CI: confidence interval; ADL: activities of daily living; IADL: instrumental ADL; pp: percentage points.

Table S11. ENIGH 2022 disability prevalence and sensitivity of the catastrophic-expenditure association to the disability threshold.

A. Survey-weighted disability prevalence under alternative ENIGH thresholds (inverted scale)

Definition	Prevalence % (95% CI)	Comparator (Census/INEGI)
Strict: “cannot do it” only ($=1$)	1.26 (1.20–1.32)	—
Primary: great difficulty or worse (≤ 2)	6.75 (6.59–6.92)	5.4–5.7% (Washington Group)
Broad: some difficulty or worse (≤ 3)	16.41 (16.12–16.70)	16.5% (any disability/limitation)

The primary ENIGH definition (≤ 2) yields a prevalence (6.8%) closely matching the Washington Group estimate from the Census (5.4–5.7%), and the broad definition matches the national “any disability or limitation” figure (16.5%), empirically confirming the inverted scale and the alignment of the ≤ 2 threshold with the Washington Group criterion. These are *individual*-level disability prevalences, reported here to validate the inverted-scale coding; the catastrophic-expenditure models in main-text Table 4 and in Panel B below instead use a *household*-level exposure (at least one resident member with disability), which by construction covers a higher proportion of households than the individual prevalence.

B. Fully adjusted odds ratio for catastrophic health expenditure (PWD household), by disability threshold

Definition	Primary threshold (≤ 2)	Strict threshold ($=1$)
WHO (>40% capacity to pay)	2.02 (1.73–2.35)	2.44 (1.89–3.15)
Alternative (>10% income)	1.96 (1.78–2.16)	2.27 (1.95–2.64)

Survey-weighted logistic regression (quasibinomial), fully adjusted for income quintile, household size, presence of an elderly member, and rural residence (M3 specification, matching main-text Table 4). Under the strict threshold the household disability prevalence falls roughly five-fold, yet the catastrophic-expenditure odds ratio is, if anything, larger, indicating the association is not an artifact of an inclusive disability definition. PWD: persons with disabilities; WHO: World Health Organization; CI: confidence interval.

Table S12. Survivor- and selection-bias sensitivity analyses for the prospective mortality models.

A. E-values (VanderWeele & Ding) for key associations

Association (fully adjusted)	OR (95% CI)	E-value (point)	E-value (CI bound)
Disability → mortality	3.77 (3.02–4.70)	3.30	2.87
Non-contributory SS → mortality	0.89 (0.71–1.12)	1.31	1.00
Unaffiliated SS → mortality	1.11 (0.80–1.51)	1.29	1.00

B. Stability of associations in less selection-prone subgroups

Analytic sample	N (deaths)	Disability OR	Non-contributory SS OR
Full M4 cohort	10,169 (498)	3.77 (3.02–4.70)	0.89 (0.71–1.12)
Excluding severe baseline disability	9,944 (423)	2.77 (2.13–3.59)	0.90 (0.71–1.14)
Excluding baseline chronic disease	4,855 (108)	4.39 (2.47–7.50)	0.97 (0.60–1.58)

Individuals who died before the 2018 baseline are necessarily excluded (left truncation), which would tend to bias the non-contributory social-security association toward the null. Panel A reports E-values: the disability–mortality association is highly robust (an unmeasured confounder would need to be associated with both exposure and mortality by a risk ratio of 3.30, or 2.87 for the lower confidence bound, to explain it away), whereas the non-contributory association is fragile (E-value 1.31; confidence interval already includes the null), consistent with susceptibility to residual selection. Panel B shows that the null non-contributory association is stable when the groups most affected by survivor selection (severe baseline disability; baseline chronic disease) are excluded, indicating it is not driven solely by differential early mortality. Together these analyses probe the robustness of the null non-contributory association under two indirect sensitivity checks rather than directly quantifying the magnitude of left truncation, and they motivate the cautious, non-causal interpretation adopted in the Discussion. OR: odds ratio; SS: social security; CI: confidence interval.

Table S13. Fit statistics of the nested survey-weighted logistic models for lack of social security affiliation (M1–M5), Census 2020.

Model	Covariates added	N	Residual deviance	Pseudo-R^2
M1	Disability (unadjusted)	10,739,396	11,539,454	0.0004
M2	+ sex, age group	10,739,396	11,466,449	0.0067
M3	+ education	10,717,409	11,359,142	0.0132
M4	+ indigenous, rural	10,717,409	11,348,643	0.0141
M5	+ disc$\times$sex, disc$\times$indigenous	10,717,409	11,348,362	0.0141

Survey-weighted logistic regression (quasibinomial). Because quasi-likelihood survey-weighted models do not admit a standard Akaike Information Criterion, model fit is summarized by residual deviance and the McFadden pseudo-R^2 (1 − residual/null deviance); both improve progressively across specifications. The key evidence on confounding, however, is the trajectory of the *disability* odds ratio itself (main-text Table 2): it moves from 0.82 (M1) to 0.99 after adjustment for age and sex (M2), indicating that the apparent aggregate coverage advantage is essentially fully explained by the older age structure of PWD; education and the remaining covariates produce only minor further change. Absolute pseudo-R^2 values are low, as expected when modeling a population-scale binary outcome from a small set of sociodemographic covariates. Residual-deviance values match those reported in main-text Table 2 and Supplementary Table S9. N differs between M2 and M3 owing to 21,987 records with missing education.

Table S14. Nested logistic regression models for prospective three-year mortality, ENASEM 2018→2021.

Model	Covariates	OR	95% CI	*p*	AIC
M1	Disability only (N = 10,268)	6.14	5.03–7.47	<0.001	3,725
M2	+ Age, sex (N = 10,268)	4.60	3.70–5.70	<0.001	3,473
M3	+ Education (N = 10,169)	4.44	3.56–5.51	<0.001	3,456
M4	+ SS type, chronic conditions (N = 10,169)	3.77	3.02–4.70	<0.001	3,384
M5	Severity gradient (M4 covariates)	—	—	—	3,361
Mild (IADL only)		1.89	1.07–3.15	0.020	
Moderate (1–2 ADL)		3.14	2.37–4.14	<0.001	
Severe (3+ ADL)		7.24	5.18–10.0	<0.001	

M = nested multivariate logistic regression model. OR = disability odds ratio. M5 replaces binary disability with 4-level severity (ref: no disability). N decreases from M2 to M3 owing to complete-case requirements for education (99 records with missing education data). Full M1–M4 coefficients are in Table S8; risk ratios for these models are in Table S10; the progressive attenuation is visualized in Figure 3. OR: odds ratio; CI: confidence interval; SS: social security; AIC: Akaike Information Criterion; ADL: activities of daily living; IADL: instrumental ADL.

Figure S1. Disease prevalence by disability status

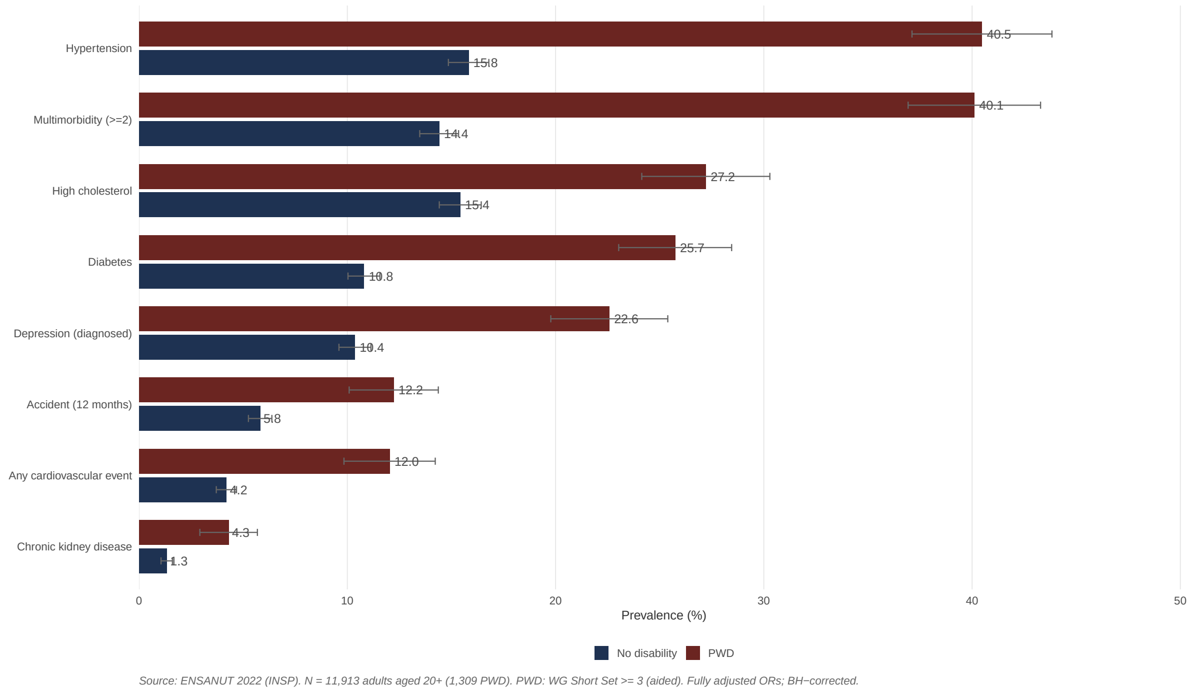

Survey-weighted crude prevalence (not model-adjusted) of eight physician-diagnosed conditions among adults aged 20+ years, stratified by disability status. Error bars represent 95% confidence intervals. These are descriptive comparisons; age- and sex-adjusted ORs are reported in Table 6 and Figure S2. All comparisons significant at $p < 0.001$ after BH correction. Source: ENSANUT 2022 (INSP). N = 11,913 (1,309 PWD). PWD: persons with disabilities; WG: Washington Group; BH: Benjamini–Hochberg; CI: confidence interval.

Figure S2. Adjusted odds ratios for the assessed conditions: PWD vs. no disability

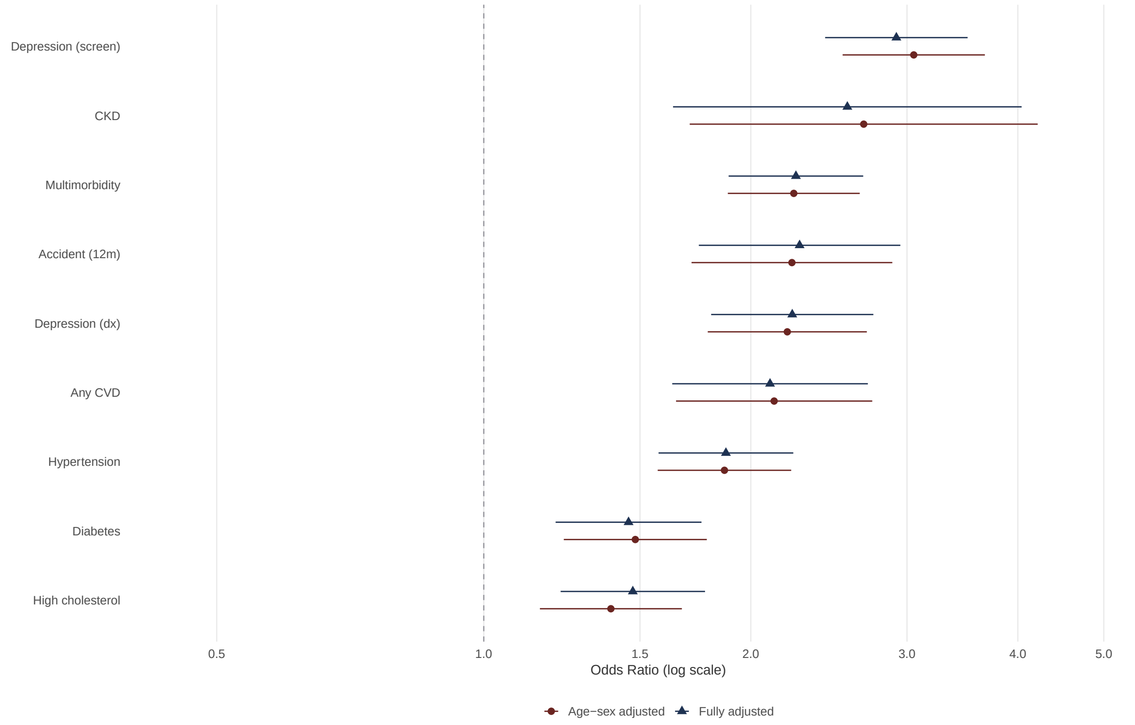

Forest plot comparing age-sex adjusted (circles) and fully adjusted (triangles; age + sex + education + indigenous language) odds ratios for nine conditions. Full adjustment attenuated ORs by $<5\%$ for all conditions, suggesting disability is independently associated with these conditions after accounting for age structure and socioeconomic position. Dashed line at OR = 1.0. All $p_{\text{adj}} < 0.001$ after BH correction. Source: ENSANUT 2022 (INSP). N = 11,913 (1,309 PWD). OR: odds ratio; CI: confidence interval; BH: Benjamini–Hochberg; PWD: persons with disabilities.

Figure S3. Inter-wave mortality rate by age group and sex, ENASEM 2018–2021

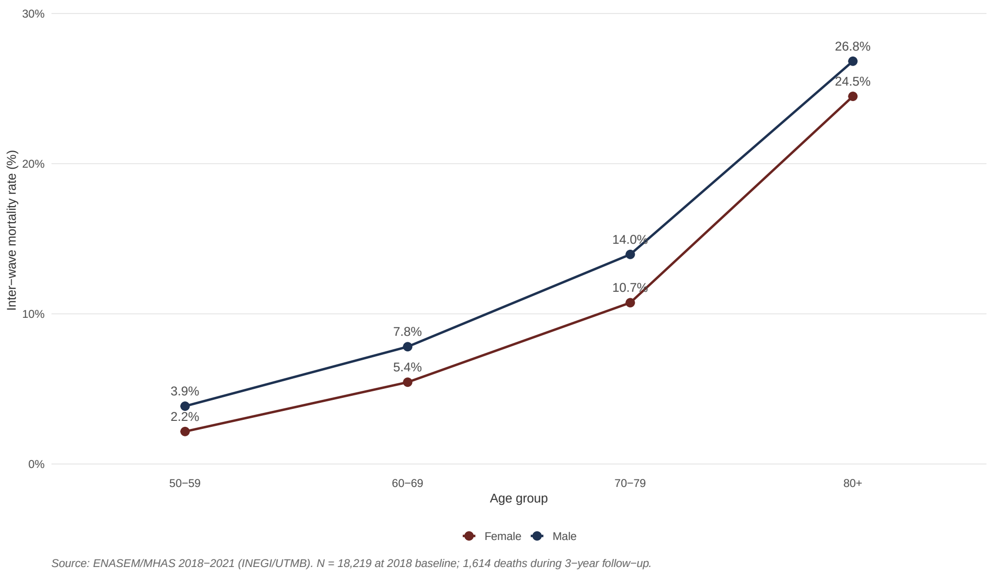

Three-year mortality rates among adults aged 50+ at 2018 baseline, showing strong age and sex gradients. Males had consistently higher mortality across all age groups. Source: ENASEM/MHAS 2018–2021 (INEGI/UTMB). N = 18,219 at baseline; 1,614 deaths. ENASEM: Estudio Nacional de Salud y Envejecimiento en México; MHAS: Mexican Health and Aging Study.

Figure S4. Triple vulnerability among PWD by state (dot plot)

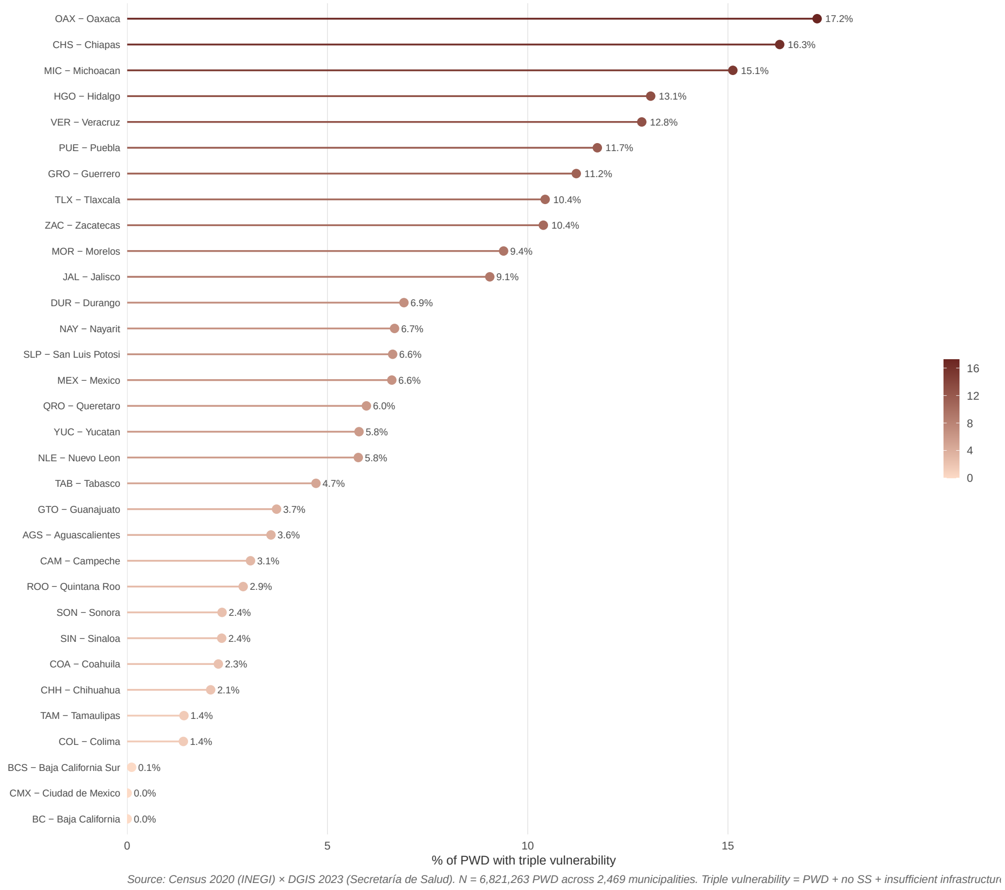

Proportion of PWD in each state who are simultaneously unaffiliated with social security and reside in municipalities with insufficient healthcare infrastructure ($\leq$30 hospital beds). Dot plot version of the choropleth map in Figure 4B of the main text. Source: Census 2020 (INEGI) $\times$ DGIS 2023 (Secretaría de Salud). N = 6,821,263 PWD across 2,469 municipalities. PWD: persons with disabilities; SS: social security; DGIS: Dirección General de Información en Salud.
